# Sustainable proteins for future food systems: nutritional quality, environmental trade-offs, and consumer acceptance

**DOI:** 10.3389/fnut.2026.1913634

**Published:** 2026-08-10

**Authors:** Sadaqat Ali, Benish Ali, Khurshid Ahmad, Li Zhang, Qazi Mohammad Sajid Jamal

**Affiliations:** 1 College of Food Science and Engineering, Guangdong Ocean University, Zhanjiang, Guangdong, China; 2 Department of Orthotics and Prosthetics, Government College University, Faisalabad, Pakistan; 3 Department of Health Informatics, College of Applied Medical Sciences, Qassim University, Buraydah, Saudi Arabia; 4 College of Coastal Agricultural Sciences, Guangdong Ocean University, Zhanjiang, Guangdong, China

**Keywords:** alternative protein sources, circular economy, consumer acceptance, environmental footprint, food system transition, life cycle assessment, nutritional quality, sustainable protein

## Abstract

The global food system is under growing strain to deliver sufficient high-quality protein for a rising population, while curbing environmental degradation, tackling climate change, and maintaining long-term food security. Conventional livestock production is a major source of greenhouse gas emissions, excessive land and water use, and biodiversity loss, which has boosted demand for sustainable alternative proteins such as plant-based, microbial, algal, insect, fermented, and cultivated meat products. This multidisciplinary review comprehensively assesses these emerging protein sources across nutrition, environmental performance, and consumer acceptance. It evaluates their amino acid composition, protein digestibility, micronutrient density, and health effects, and quantifies their environmental impacts using life cycle assessment indicators. Noticeable trade-offs in processing intensity, energy consumption, and production scalability are also discussed. Key factors shaping consumer adoption, including sensory traits, cultural perceptions, affordability, and food neophobia, are analyzed. This study further explores technological advances, circular economy strategies, regulatory updates, and policy support for protein transition, identifies critical research gaps, and proposes an integrated multi-dimensional evaluation framework. The findings provide practical guidance for researchers, industrial practitioners, and policymakers to build resilient, equitable, and eco-friendly protein systems benefiting both human and planetary health.

## 1 Introduction

The global food system currently stands at a critical juncture, facing the dual challenge of nourishing an expanding human population while operating within the stringent ecological boundaries of our planet. The world population is expected to reach about 9.8 billion by 2050, and the need for key nutrients like protein is projected to greatly increase (1). This demographic shift is compounded by an aging world population that demands increased protein in the diet for maintaining musculoskeletal function and metabolism. Global consumption of meat is estimated to grow by almost 70% in the next 30 years if the current trend continues. The increased demand is not just a logistical challenge but a direct threat to environmental balance, since the livestock farming system is very resource-consuming. Currently, around 50% of the habitable land is used for agriculture, and it produces around 30% of anthropogenic greenhouse gases (GHGs), primarily from livestock production. Furthermore, nearly 70% of global freshwater withdrawals are attributable to this industry, which leads to water security issues and ecological damage in many regions (2). Intensive livestock farming also requires the use of synthetic fertilizers and pesticides that potentially result in soil acidification and biodiversity loss. Apart from the environmental concerns, animal-based proteins have also been linked to elevated risks of obesity and type 2 diabetes, as well as cardiovascular diseases in many countries (3). The food system today is therefore becoming increasingly unsustainable, not only because of the increasing nutritional demand but also because the system cannot sustain ecosystem health. The idea of a protein transition has become a key future food security issue to address these systemic vulnerabilities. This transition is characterized as a shift to a more diversified and sustainable protein supply, producing and consuming fewer animal-source foods (4).

Generally, sustainable proteins are those which deliver similar or better nutritional value and substantially lower environmental impacts; some alternatives can achieve up to 70% less environmental impact than standard beef production (5). These new protein sources are generally divided into four main groups—plant-based proteins, microbial proteins, insect proteins, and cell-cultured meat. The most widely known class are plant-based proteins, which are found in legumes, pulses, and mushrooms, and have known health benefits and lower production costs (6). Microbial proteins like single-cell proteins (SCPs) and mycelium are manufactured by means of precision or biomass fermentation, which can be done separately from climate conditions and have high land-use efficiency (7). Insect proteins, like crickets or mealworms, are of high quality and contain all essential amino acids, and can be fed with organic waste streams, which promotes a circular bioeconomy. Finally, cell-cultured or lab-grown meat represents a new technology frontier which attempts to recreate the sensory and nutritional value of traditional meat by growing animal cells in bioreactors, with the potential to save the lives of animals and the space traditionally used for grazing (1).

The incorporation of these sustainable proteins is not just a technical imperative but also a direct action toward the United Nations Sustainable Development Goals (SDGs), specifically Goal 2 (Zero Hunger), Goal 6 (Clean Water and Sanitation), Goal 12 (Responsible Consumption and Production), and Goal 13 (Climate Action). Alternative proteins can help facilitate this transition toward a more resilient and equitable global food system by removing the need for intensive land and water cultivation. This harmony is echoed by the principles of the “Planetary Health Diet,” which recommends a diet composed primarily of plant-based and alternative proteins to ensure the health of the planet and its inhabitants (4). However, achieving these global objectives involves overcoming large regional differences. Most of the alternative protein research and corporate investment is now focused in high-income countries, but the fastest growth in protein demand and the greatest environmental strain from ruminant livestock is projected to come from developing regions by 2050. Thus, any successful transition must be inclusive, meaning alternative proteins must be affordable and culturally appropriate across various socio-economic statuses, and must also take into account the livelihoods of the 1.3 billion people currently relying on the livestock sector (8).

However, there are complex trade-offs in the uptake of sustainable proteins that need to be well managed, even though there are clear environmental benefits. A key issue is the nutritional profile and bioavailability of alternatives to animal protein. Many alternatives are high in fiber and low in saturated fats but may lack certain micronutrients such as vitamin B_12_ or iron, and some meat alternatives are highly processed, which can raise sodium levels. Furthermore, the production and manufacturing of these proteins can also have environmental costs, such as the energy-intensive processes involved in cell-cultured meat and precision fermentation. Consumer acceptance is likely to be the greatest challenge. Another significant constraint on market entry is neophobia, defined as “the fear of novel foods,” as well as cultural preferences for traditional meat and the sensory characteristics of alternatives (9). Beyond product affordability, price parity with conventional meat is another key precondition for mass adoption, which was not achieved by the vast majority of the world's population in recent years (9). The objective of this review is to provide a comprehensive analysis of the current landscape of sustainable proteins, evaluating them through the lenses of nutritional quality, environmental trade-offs, and the psychological and socio-economic drivers of consumer behavior. By synthesizing the latest research across these three dimensions, this review aims to identify the pathways toward a truly sustainable and acceptable future food system. Previous reviews have synthesized aspects of alternative protein research, including systematic reviews of consumer acceptance interventions (10) and meta-reviews of meat reduction behavior (11). The present review offers a novel contribution by integrating the nutritional, environmental, circular economy, consumer acceptance, economic, and technological dimensions of the alternative protein transition within a single multidisciplinary framework, rather than treating them in isolation. It is intended primarily as a resource for researchers designing future empirical or life cycle studies, for food industry decision-makers evaluating investment and product development priorities, and for policymakers shaping regulatory and labeling frameworks.

## 2 Review methodology and scope

This review adopts a narrative, multidisciplinary synthesis rather than a systematic review format, consistent with recognized typologies of literature reviews (12). Relevant literature was identified through structured searches of Scopus, Web of Science, PubMed, and Google Scholar for the period 2015–2026, using combinations of keywords including “alternative protein,” “plant-based meat,” “cultured meat,” “insect protein,” “microbial protein,” “consumer acceptance,” “food neophobia,” “life cycle assessment,” and “circular economy.” Searches spanned food science, nutrition, environmental science, agricultural economics, and consumer/social psychology disciplines and were supplemented by manual screening of reference lists of key reviews. Articles were prioritized for inclusion based on relevance to the nutritional, environmental, circularity, or consumer acceptance dimensions of alternative proteins, recency, and publication in peer-reviewed journals. Non-English sources and non-peer-reviewed gray literature were excluded except where cited for market or regulatory context. Given the breadth and heterogeneity of the disciplines and outcome measures covered, a systematic review with quantitative synthesis was not undertaken; instead, this narrative review qualitatively synthesizes converging evidence and flags areas of disagreement or limited evidence throughout the text.

## 3 Sustainable protein sources: current landscape

### 3.1 Plant-based proteins

**Figure 1:**
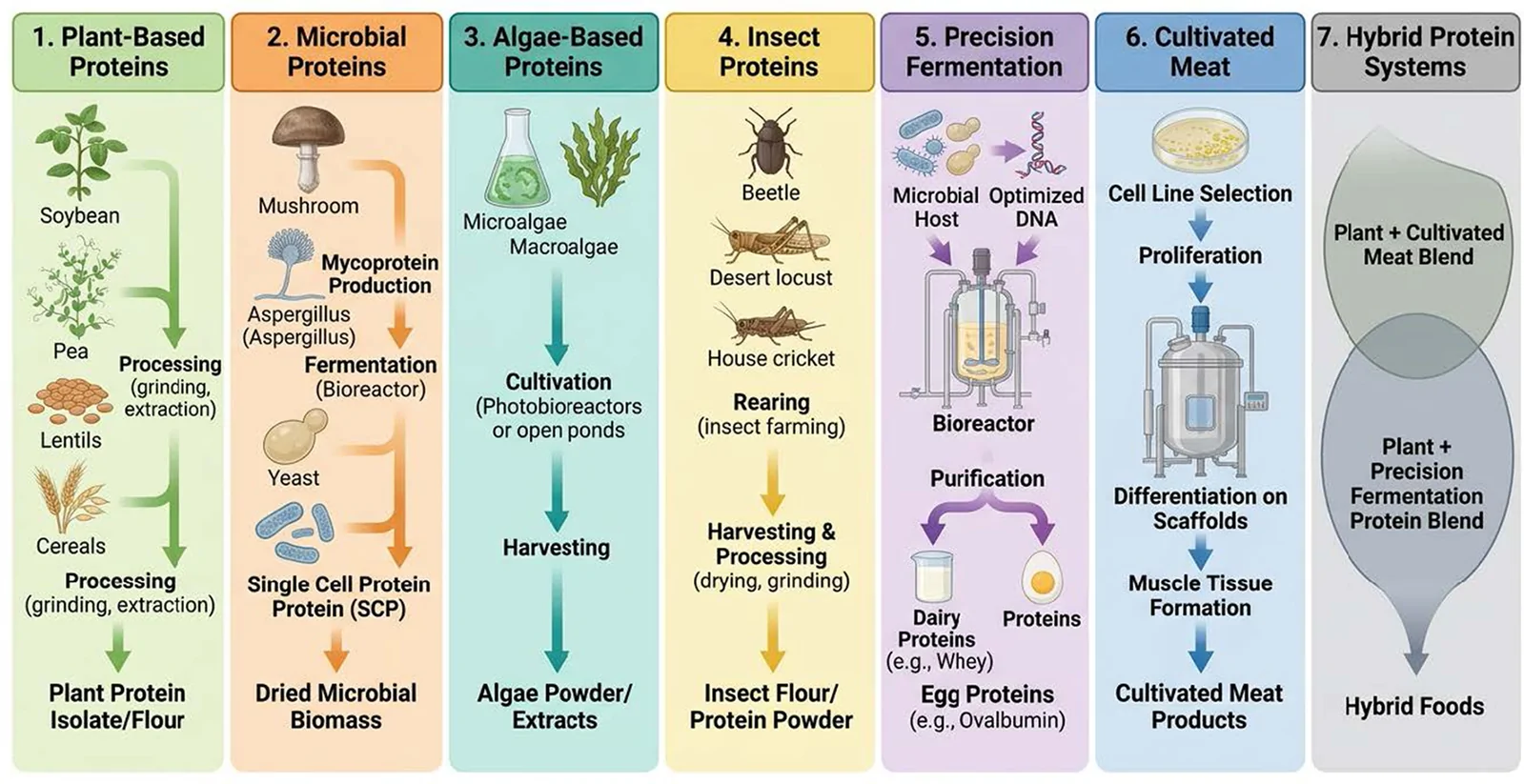
Classification and production pathways of sustainable protein sources. Created with BioRender.com.

The plant-based protein sector is the most developed and widely used in the alternative protein arena, largely due to its existing farming systems and consumer awareness (Figure 1). Soybean is undisputedly at the top of this field because of its remarkable amino acid profile and high Protein Digestibility Corrected Amino Acid Score (PDCAAS) compared with some animal proteins. The functionality of soy, such as forming fibrous textures in the extrusion process, is utilized for producing meat analog products that mimic the mouthfeel of conventional meat products (13). By contrast, the carbon footprint of soy-based alternatives is significantly lower, by a factor of 4 to 20, and they have high resource efficiency when compared to beef (14). Pea protein is the next most popular ingredient for meat substitutes and has also gained popularity as a next-generation ingredient. Pea protein is especially recognized for its high quality and hypoallergenic nature, making it an excellent option for the growing gluten-free and soy-free market sectors (15). It can be added to a variety of products, including plant-based milk, 3D-printed meat structures, and other applications, with minimal water usage and low environmental impact (16).

In addition to these major isolates, the larger group of lentils and other legumes are also important for human nutrition and agriculture. These crops are naturally enriched in dietary fiber and minerals like iron and zinc, which are essential to combat the hidden hunger normally linked to grain-heavy diets. Legumes are irreplaceable in ecological terms because they have a special ability to fix nitrogen biologically, which enriches the soil and thus saves on the use of chemical fertilizers, thereby favoring wider agro-biodiversity (17). In addition, cereals, such as wheat, are often used to meet structural needs and in combination with legumes to satisfy the gluten requirement, which is essential for the viscoelasticity and chewiness of many meat analogs. To address the nutritional deficiencies of single plant-derived products, such as the lack of lysine in cereal grains, the industry is increasingly turning to pseudocereals, like quinoa and amaranth. Pseudocereals are widely recognized as a good source of a complete amino acid profile, serving as a nutritional bridge that facilitates the provision of plant-based diets to satisfy all human physiological needs (18). The current landscape relies upon an intricate approach to protein complementation and fortification, pairing a variety of plant-based proteins for both sensory and nutritional benefits, so that the shift to plant-based food systems is as nutritious as it is sustainable (19).

### 3.2 Microbial proteins

Microbial proteins, often referred to as single-cell proteins (SCPs), are a promising step forward in the quest for sustainable food systems. They utilize microorganisms to produce nutrient-rich proteins from a variety of raw materials (20). These proteins can be sourced from bacteria, yeast, fungi, and microalgae, offering a scalable alternative to traditional farmed protein production, which is often associated with environmental degradation and high resource requirements (21). The main benefit of SCPs is their remarkable resource efficiency, requiring significantly less land and water than animal agriculture. They can be produced in high-yield bioreactors, without seasonal or regional constraints. Moreover, microbial biomass can be produced as part of a circular economy, using agro-industrial by-products or renewable gases as inexpensive raw materials, thereby minimizing waste generation and greenhouse gas emissions. While the high energy demand of a sterile bioreactor is a compromise, the significant reduction in land use and the opportunity to use non-arable land offer a more positive ecological balance than conventional farming practices (22).

A prominent subset of this landscape is mycoprotein, produced through the fermentation of filamentous fungi, most notably *Fusarium venenatum*. Mycoprotein is well recognized for its excellent nutritional profile, with high protein content (up to 71% dry matter), a full range of essential amino acids, and significant levels of dietary fiber from 4.8% to 25% (23). It has a significantly lower environmental impact than beef, with greenhouse gas emissions reduced by over ten times, supporting the shift toward more sustainable diets. In addition to its ecological impact, mycoprotein intake has shown clinical advantages in muscle protein synthesis and favorable cardiometabolic health markers such as better lipid profiles and lower cholesterol (24). The increased fiber also contributes to gut health, providing a functional health benefit alongside its protein source. Furthermore, yeast proteins are also of great importance in the present scenario, especially in the formulation of meat substitutes and functional food ingredients (21). Yeasts such as *Saccharomyces cerevisiae*, cultivated by either liquid or solid fermentation, are most commonly used due to their safety profile, with many strains designated as Generally Regarded as Safe (GRAS). These proteins can be used as a source of essential amino acids and are increasingly produced through biorefinery concepts, which transform food waste into value-added products (25). Likewise, bacterial proteins are highly metabolically flexible, some of which can synthesize high-quality biomass using methane or carbon dioxide. In gas fermentation processes, protein production is completely independent of traditional agriculture, and industrial waste gases are used to produce proteins with high digestibility and low land requirements. These bacterial sources help to reduce the environmental pollution and methane emissions of conventional protein production by replacing land-based, cattle-intensive production systems with managed microbial systems (20).

### 3.3 Algae-based proteins

Algae are a key frontier in blue food systems as a high-density protein source that can avoid land and freshwater constraints of terrestrial agriculture. These aquatic organisms are mainly classified into microalgae (microscopic unicellular organisms) and macroalgae (seaweeds), offering different nutritional and functional values for future food security. Microalgae, particularly *Chlorella* and *Arthrospira* (*Spirulina*), have been noted for their high protein content, which can range from 50 to 60% of dry biomass (26). Their amino acid composition is similar to high-quality animal proteins, with all essential amino acids (EAAs) present in proportions that meet or exceed WHO/FAO requirements. In addition to protein, microalgae contain high levels of bioactives such as antioxidants, carotenoids, and omega-3 fatty acids (DHA/EPA), which support gut and liver health (27). One serious obstacle to their broad application is that they are embedded in recalcitrant cell walls, which might restrict their bioavailability in the human digestive tract. Consequently, advanced processing techniques such as high-pressure homogenization or enzymatic hydrolysis are increasingly employed to disrupt these structures and enhance protein digestibility (28). Macroalgae (or seaweeds) are more variable protein sources, such as *Porphyra, Ulva*, and *Palmaria*. Their protein content varies from 5 to 47% depending on the species and season, but in some species, protein content is similar to legumes. Macroalgae are particularly rich in minerals (iodine, calcium, magnesium) and dietary fibers (29). In terms of sustainability, macroalgae cultivation is very efficient as they do not need any fertilizers since they absorb nutrients directly from the marine environment, thus also helping to lower coastal eutrophication (28). Algal proteins also possess functional versatility, such as emulsifying, gelling, and foaming capabilities, which has inspired their incorporation into different food matrices. Microalgal biomass is also being utilized as a key component in the development of meat analogs to mimic the fibrous structure and savory flavor of traditional meat, while also providing a complete protein source (30). In addition, algae are added to baked goods like protein-enhanced bread and snacks, contributing to their nutritional value and functional shelf life. They are also the main source for vegan omega-3 and bioactive peptides with antihypertensive properties. However, sensory characteristics such as a fishy taste and dark green color are still consumer barriers to market acceptance, and innovations in flavor masking and color stabilization are needed to increase consumer acceptance (31).

### 3.4 Insect proteins

Insect protein is becoming a key ingredient in the sustainable protein equation with its nutrient composition and environmental benefits compared to conventional livestock. Currently, only a few species dominate the commercial market, among them the yellow mealworm *Tenebrio molitor*, the house cricket *Acheta domesticus*, and the black soldier fly *Hermetia illucens*, which is especially appreciated for its high bioconversion efficiency. These insects are highly proteinaceous, generally ranging from 30 to 85% protein (dry matter basis), and are a rich source of all essential amino acids, which can be higher than traditional protein food sources such as beef and chicken (32). In addition to macronutrients, insects are also rich in beneficial lipids, such as omega-3 and omega-6 polyunsaturated fatty acids, and bioavailable micronutrients, including iron, zinc, and B-vitamins, which makes them a powerful solution to global nutritional security (33). Insect farming is part of a more sustainable production system known as the circular bioeconomy and is a pillar of the circular bioeconomy. In contrast to conventional livestock, insects can be raised using organic side-streams and agricultural wastes, and the biomass is effectively used and valorized as high-value protein (34).

This offers a great benefit in the environmental footprint of food production: up to 100 times less GHS is emitted than beef, and up to 50%−90% less land and water is used than for beef (35). Technological advancements, such as AI-driven climate control and automated harvesting, enhance economic viability and scalability while reducing resource requirements, including soybean and fishmeal (36). Yet, there are some regulatory and safety issues with the widespread use of insect proteins. The European Food Safety Authority (EFSA), under the Novel Food Regulation, conducts a comprehensive safety assessment in the European Union for insect products, including determining potential microbial contamination, bioaccumulation of heavy metals, and cross-reactivity in individuals with shellfish sensitivity. While several species have already received positive food safety opinions, the criteria for labeling and food-grade production of these species differ by jurisdiction (37). These international frameworks need to be harmonized to ensure consumer protection while facilitating trade. Additionally, the cultural disgust factor is a crucial hurdle, but processing insects into unrecognizable forms, like powders, isolates, and pastes, and adding them to familiar food matrices such as pasta, snacks, and meat analogs has been found to be promising in enhancing consumer acceptance (38).

### 3.5 Precision fermentation proteins

Precision fermentation (PF) is one of the most important pillars of acellular agriculture, where specialized animal-identical proteins are produced without relying on conventional livestock rearing (39). The technology is based on the fundamental principles of synthetic biology, in which the gene coding for an animal protein (e.g., milk or egg protein) is transferred to a host cell (e.g., yeast, bacteria, or filamentous fungi) (40). The genetically engineered microorganisms are used as biological factories in a controlled bioreactor, expressing target proteins through a very efficient fermentation process by feeding them simple feedstock such as glucose. After production, the microbial biomass is isolated, and the proteins are purified to obtain a product indistinguishable at the molecular level from those obtained from animal sources (41). This precision allows for the production of clean ingredients with the precise amino acid sequences and structural properties needed for traditional uses in the food industry. PF is being used in the dairy industry for manufacturing important milk proteins such as whey (beta-lactoglobulin), casein, and valuable milk proteins such as lactoferrin. The recombinant proteins offer identical nutrient profiles (unlike plant-based products) and functional properties, including melting and stretching, which are critical for cheese manufacturing (42). Likewise, the technology has been successfully applied to target egg proteins, and ovalbumin, the main protein of egg white, is already being produced with fungal hosts such as *Trichoderma reesei*. This recombinant ovalbumin can be used directly as a functional substitute for chicken egg white powder for its foaming, gelling, and binding properties in the bakery and confectionery industry (43). In addition to nutritional benefits, precision fermentation is opening the door to functional protein ingredients, such as animal-free gelatin and specific enzymes. PF can overcome sensory and textural drawbacks often encountered in the mass adoption of plant-based alternatives by supplying molecular replicas of animal proteins (44). Furthermore, this production model has clear environmental benefits, as protein is not produced using resource-intensive ruminant farming. Proteins made through PF have the potential to significantly lower land use and GHG emissions, with the final environmental cost depending on the energy profile and carbon sources during the industrial process (45). Scaling up the technology will yield a resilient, sustainable source of high-quality proteins, meeting the needs of industry and changing consumer demand for ethical food systems (46).

### 3.6 Cultivated meat

Cultivated meat is a paradigm shift in food production, using cellular agriculture to create animal tissue without traditional animal farming. The process starts with the selection of high-quality cell lines, usually adult stem cells like muscle satellite cells or pluripotent cells like induced pluripotent stem cells (iPSCs), that have the ability to self-renew and differentiate into muscle and fat (47). To replicate the complex three-dimensional architecture of conventional meat, these cells are seeded onto biocompatible scaffolds made from edible or biodegradable biomaterials. The scaffolds provide mechanical and biochemical support to guide cell attachment and tissue maturation, resulting in a final product with a texture and structure similar to traditional meat products (48). Advanced bioprocessing platforms support the scale-up from laboratory experiments to industrial-scale products. Large-scale production is performed in well-designed bioreactors, which enable environmental control, e.g., temperature, pH, and oxygen availability, for optimal cell growth (49). One major research interest has been the formulation of serum-free, chemically-defined culture media to substitute for animal-derived supplements, such as fetal bovine serum, which are high in cost and have raised ethical concerns (50). Media costs have been significantly reduced, but economic parity with traditional livestock has been a big challenge. The scale-up to bioreactors with volumes > 50 liters poses new technical problems such as metabolic waste control, nutrient distribution, and cell viability. Cultivated meat is commonly touted as having the potential for significant reductions in land and water use compared to industrial livestock production from an environmental standpoint (51). Recent life cycle assessments indicate that this benefit depends on renewable energy usage since bioreactor operating processes have high energy requirements that can otherwise lead to high carbon footprints. Nutritionally, the goal is to replicate or improve the nutrient profile of conventional meat; however, there are still some gaps in the scientific understanding of the long-term health effects and the specific bioavailability of micronutrients in these new products (52). Commercialization of cultivated meat is in an early stage with significant investment and few advances in regulations. While there are no consistent international systems in place to enable market access, certain countries have provided early approvals for specific products, such as Singapore and the United States. Consumers are another major challenge as they tend to be discouraged by neophobia and doubts about lab-grown foods (53). Nevertheless, the industry is moving forward, and over 170 companies are working to test the potential of integrating cellular innovations with scalable technology to create a more resilient and sustainable food system of the future (54).

### 3.7 Hybrid protein systems

The transition to a sustainable food system requires a pragmatic strategy, such as hybrid protein systems that combine conventional animal proteins with alternative proteins like plants, insects, or microorganisms (55). In general, these dual protein products are developed by substituting 20 to 50% of the animal matrix with another ingredient to reduce the negative impact of livestock production without losing the sensory and nutritional characteristics appreciated by consumers (56). The most prevalent hybrid combinations are plant-animal products, which involve the use of functional plant ingredients, such as faba bean flour, broccoli, or upcycled brewer's spent grain, in meat and dairy products. The sensory parity of traditional and analog sausages and spreadable cheeses has been demonstrated for some critical parameters, including juiciness, odor, and mouthfeel, across a variety of spreadable and dry sausage styles (57). This blended approach is a strategic solution for flexitarian consumers who might not be satisfied with a food made entirely from plants because of inconsistencies in texture and/or taste (58). Technical challenges still remain, however: high substitution levels can introduce unwanted beany or earthy flavors, and protein-protein interactions must be carefully controlled by pH and temperature to prevent degradation of color and texture. Next-generation hybrid products build on this idea and feature proteins from precision fermentation or biomass fermentation with a variety of plant-based bases (59). These cutting-edge systems utilize cellular agriculture to manufacture animal-identical molecules like recombinant whey or casein which, coupled with plant-derived fats and fibers, are used to formulate complex food architectures. Technologies like 3D printing, high-moisture extrusion, and high-pressure processing are key in structuring these plant-fermentation combinations to more closely mimic the organoleptic properties of meat and dairy than a simple botanical blend (60). Nutritionally, hybrid systems offer an optimal nutritional profile with well-defined essential amino acid scores, digestion, and protein bioavailability. In addition, these products tend to be associated with better fatty acid profiles, as well as increased fiber and decreased saturated fat and cholesterol compared to traditional animal products (61). Environmental trade-offs favor hybrid systems because the land, water, and GHG intensity of ruminant and poultry farming is significantly lower in the hybrid approach. While the use of alternative ingredients does consume energy, the overall environmental impact is still significantly lower than in traditional livestock systems (59). Consumer acceptance of these hybrid solutions is complex and relates significantly to health perceptions and sensory familiarity. Consumer choice is driven by perceptions of healthiness, although environmental and animal welfare concerns are influential. Thus, hybrid protein systems can serve as a low-threshold roadmap for dietary transition and provide a scalable and scientifically sound approach toward achieving a more sustainable global food system (58). It should be noted that consumer choice in this context is shaped by a considerably broader set of factors than healthiness, environmental impact, and animal welfare alone, including familiarity, perceived naturalness, technological literacy, social norms, and perceived risks and benefits, several of which can act simultaneously as drivers and barriers rather than falling neatly into either category (62, 63).

## 4 Nutritional quality of sustainable proteins

### 4.1 Protein quality assessment methods

Evaluating the nutritional quality of alternative proteins is fundamental to the global transition toward sustainable food systems. These proteins must meet human physiological requirements in the same way as conventional animal proteins. The basic approach to this assessment has been the Protein Digestibility Corrected Amino Acid Score (PDCAAS), which measures protein quality based on the ratio of a food's amino acid composition to a reference pattern, corrected for protein digestibility in the feces (64). Although PDCAAS has been a standardized approach for decades, it has limitations and can be difficult to apply to new and sustainable proteins. Its dependence on fecal nitrogen analysis may lead to an overestimation of protein absorption because of the metabolic activity of colonic microflora (65). Moreover, truncating PDCAAS at 100% obscures the superior nutrition of high-quality proteins (HQPs), which can be used in conjunction with low-quality plant proteins (LQPs). To overcome these limitations, the Digestible Indispensable Amino Acid Score (DIAAS) has been adopted as the metric of choice. DIAAS uses ileal digestibility to better reflect the protein available for absorption in the small intestine (66). DIAAS is especially important for assessing sustainable proteins such as microalgae, which contain nitrogenous components other than protein, or chitin in insect exoskeletons, which can cause significant errors if not corrected using standard nitrogen-to-protein conversion factors (67). For example, mealworm larvae and crickets have shown good quality protein, but their digestibility profile is highly sensitive to processing methods (e.g., blanching or oven drying). In addition to these well-known scores, new protein quality metrics are placing greater emphasis on a holistic and metabolic approach (68). These include detailed amino acid profiling, used to determine the indispensable amino acid limitations of novel blends, allowing for the identification of suitable combinations of complementary proteins. Furthermore, technological modifications, including enzymatic hydrolysis and fermentation, are being investigated as functional metrics to enhance the bioavailability of plant proteins, which can circumvent antinutritional factors (69). Sustainable proteins are also beginning to incorporate metabolic metrics that directly measure muscle protein synthesis, allowing essential score criteria to move beyond theory to actual health effects in the future food system. These traditional and emerging metrics can be combined to optimize the nutritional efficiency of the protein transition, making sustainable diets both environmentally sustainable and nutritionally well-balanced (64).

### 4.2 Amino acid composition

Nutrient composition of sustainable proteins is inherently defined by their amino acid profile, in terms of their content and levels of EAA, as these are not produced by the human body (70). Edible insects, plant protein, and microbial biomass all have different protein profiles and can help to address human protein needs when used appropriately (71). Black soldier fly larvae (*Hermetia illucens*), house crickets (*Acheta domesticus*), and mealworms (*Tenebrio molitor*) are especially notable for their high EAA content, typically greater than 40% of the total amino acid profile. For example, cricket and locust powders have been reported to contain total EAA content twice the FAO/WHO/UNU recommendations for adult human consumption, demonstrating their potential as high-quality animal protein alternatives (72). Microbial proteins, especially those obtained from fungal or bacterial biomass, also possess good profiles, but the cell wall structure of these microbes can sometimes make the nutrients more difficult to access. Although most sustainable proteins are dense in protein, they are limited in amino acids, which are those in the smallest proportion in relation to human requirements (66). Lysine is in most cases the limiting amino acid in cereal-based plant proteins, while sulfur-containing amino acids such as methionine and cysteine are limiting in legumes (73). However, some insect proteins have specific limitations based on the insect species and processing methods; e.g., lysine and valine are limiting amino acids in black soldier fly larvae under certain drying conditions (74). It is important to recognize these limitations, as the overall nutritional quality of a protein and its capacity for muscle protein synthesis is limited by its most limiting essential amino acid. These nutritional deficiencies are overcome by using complementary protein strategies to complete the amino acid pattern. This includes mixing two or more protein sources, each with its own limiting amino acid, so that the strengths of one balance the deficiencies of the other (75). Optimized protein models based on linear programming have shown that certain combinations of plant proteins (pea, rice, canola) could even be optimized to closely mimic the amino acid profile of high-quality animal protein (whey or casein) with up to 98% accuracy. Likewise, the inclusion of insect powders in conventional cereal-based food products greatly improves the lysine content and biological value of the food. Such strategic use of amino acid complementarity is critical to ensure that sustainable diets will not be nutritionally inadequate or unhealthy (67).

### 4.3 Digestibility and bioavailability

The digestibility and bioavailability of sustainable proteins are key indicators of their nutritional value and their ability to substitute for animal proteins. The most commonly used methods for evaluating protein quality are the PDCAAS and DIAAS (76). DIAAS has been widely considered a superior indicator since it takes into account the digestibility of individual essential amino acids, instead of relying on the digestibility of total fecal amino acids, which may be affected by colonic microbial metabolism (64). The DIAAS value for conventional animal proteins like beef, eggs, and dairy is consistently high (usually > 100), whereas for sustainable options, it varies widely (77). For instance, some insect species such as mealworms and crickets have shown good *in vitro* DIAAS scores that range from 89 to 92 and are therefore considered good quality protein sources. However, unlike many plant proteins, which suffer from deficiencies in EAAs, such as lysine in cereals and methionine in legumes, these deficiencies can limit the bioavailability of plant proteins unless the EAAs are provided in strategic combinations (78). Anti-nutritional factors (ANFs) are one of the main limitations to the digestibility of plant and some insect proteins. Phytates, tannins, oxalates, and protease inhibitors are examples of phytochemicals that bind to proteins physically or chemically, thereby inhibiting their enzymatic digestion in the human gastrointestinal tract. In addition, proteins in plant tissues are commonly protected by complex cell wall matrices, which is often the case with whole grain and legume-based products (77). Several processing methods are being used to address these challenges and improve the nutritional profile of sustainable proteins. Concentrations of ANFs can also be substantially decreased upon fermentation, with fungal species such as shiitake mycelium demonstrating a positive impact on the solubility of proteins and the DIAAS of pea and rice blends. Likewise, heat treatment, enzyme hydrolysis, and high-pressure processing may help to disrupt resistant protein structures and inactivate heat-sensitive inhibitors, which may improve amino acid availability (79). However, excessive heat treatment should be avoided because it can cause undesirable Maillard reactions that permanently cross-link lysine, thus paradoxically reducing bioavailability (66). Moreover, cultured meat and mycoproteins provide a significant benefit because these sources are bioengineered to closely resemble the amino acid composition of animal tissues, without the structures of the traditional plant matrices. Ultimately, achieving nutritional parity with animal proteins requires a multifaceted approach that integrates innovative processing, precise protein blending, and advanced bioengineering to ensure that sustainable proteins can effectively meet the metabolic demands of the global population (79).

### 4.4 Micronutrient profiles

Sustainable protein sources are predominantly defined by their nutritional adequacy, including their micronutrient density and bioavailability, which can differ significantly from traditional animal-based protein sources. Traditional foods like meat continue to be an important source of highly bioavailable vitamin B_12_, heme iron, and zinc, essential for hematologic and cognitive function (80). However, while plant proteins are sustainable, they are lower in intrinsic nutrients and contain anti-nutrients such as phytates and oxalates that inhibit absorption (81). Many plant-based meat analogs therefore rely heavily on fortification to match nutrient levels with their animal-based counterparts, but the physiological utilization of these added nutrients is often less efficient than that of the nutrient in its natural form (82). For example, while legumes may achieve a high iron content, in the absence of the meat factor and the presence of non-heme iron, it is much less bioavailable than the heme iron present in red meat. New sustainable alternatives like edible insects and microalgae offer potential natural solutions to fortification. Edible insects, such as crickets and mealworms, have been found to be a source of vitamin B_12_ and calcium at concentrations similar to or higher than those in traditional livestock feed (83). In addition, the bioavailability and solubility levels of iron in some insect species are better than sirloin beef, which contradicts previous assumptions about mineral sources. Among microorganisms, microalgae are well-known for their natural bioaccumulation of zinc and calcium; certain species of microalgae are also sources of long-chain omega-3 fatty acids, especially eicosapentaenoic acid (EPA) and docosahexaenoic acid (DHA), which are usually obtained from fatty fish (84). PF and cultivated meat represent the next frontier in optimizing micronutrient profiles. By using PF, specific functional proteins like leghemoglobin can be produced to close the bioavailability gap in plant-derived products by supplying heme iron. Compared to other emerging cultivated meat products, cultivated meat has the additional theoretical benefit of its nutrient profile being engineered during manufacturing to increase calcium or omega-3 content (85). However, providing consistent and accessible nutrition from these high-tech substitutes is a major challenge. In conclusion, sustainable proteins are an important part of planetary health, and a shift toward these types of proteins should consider the natural abundance and bioavailability of essential micronutrients to avoid global deficiencies of iron, zinc, and vitamin B_12_ (86).

### 4.5 Functional and health-promoting components

Besides peptides, sustainable proteins are also a source of antioxidants like phenolic compounds and carotenoids, especially abundant in plant-based and microalgae proteins. These molecules act as potent radical scavengers, mitigating oxidative stress and protecting against endothelial dysfunction, which are key drivers of cardiovascular diseases and neurodegeneration (27). The anti-inflammatory properties of these antioxidants add to the nutritional benefits of alternative protein, creating a comprehensive strategy for health promotion that is less apparent in conventional animal proteins (27). Dietary fiber and prebiotic compounds further distinguish sustainable proteins, especially those from fungal and insect sources. For example, mycoprotein has a specific cell wall matrix made of chitin and beta-glucans, which are insoluble fibers largely intact in the large intestine (87). Likewise, edible insects are rich sources of chitin, a structural polysaccharide that impacts the composition and diversity of gut microbiota. Plant proteins often come packaged with complex carbohydrates, including oligosaccharides like the raffinose family, which are fermented by beneficial gut bacteria such as *Bifidobacterium* and *Lactobacillus* (88). The synergistic action of these fibers and prebiotics leads to the production of short-chain fatty acids (SCFAs) like butyrate and propionate, which are critical for maintaining gut barrier integrity and reducing systemic inflammation. Previous studies have shown that switching to alternative protein sources can alter gut microbial composition, with a more beneficial *Firmicutes/Bacteroidetes* ratio, and improve the abundance of beneficial genera such as *Akkermansia*. Through their various activities, these functional components work together to lower levels of harmful metabolites like p-cresol and branched-chain amino acids (BCAAs), which in turn contributes to long-term metabolic health and helps lower the chance of chronic inflammatory disorders (89). The inclusion of sustainable proteins in the human diet therefore presents a strategic opportunity to take advantage of these bioactive compounds through their enhanced nutritional and therapeutic properties (27).

### 4.6 Nutritional limitations and improvement strategies

Some of the key biological challenges for sustainable protein sources, especially plant, microbe, and insect-based proteins, include unbalanced EAA profiles and the presence of ANFs such as tannins and phytates (90). To ameliorate these limitations, strategic protein blends, based on protein complementation, have become one of the main corrective actions. Amino acid sources should be optimally combined to achieve the desired PDCAAS for human physiological needs, by using lysine-rich legumes for their lysine content and cereals or pseudocereals rich in sulfur amino acids (91). For example, the amino acid profile of millet proteins has been demonstrated to be more balanced when combined with pulses, and is similar to the essential amino acid profile of animal tissues. In addition to amino acids, fortification is crucial in reducing the micronutrient “gap” of plant-based products. Bioavailable forms of iron, zinc, and vitamin B_12_ are now commonly added to many next-generation protein products so that their nutritional profile is similar to that of the traditional meat products they are intended to substitute for (92). Processing innovations are also essential in converting these raw materials into quality food ingredients. Thermomechanical treatments, especially high moisture extrusion, are widely used to achieve the fibrous and meat-like texture favored by consumers while also markedly decreasing the levels of ANFs that hinder mineral absorption and protein digestibility. In addition, fermentation is a promising bioprocessing method for nutritional improvement. Certain microbial strains can break down complex cell wall structures and enhance the solubility of proteins, as well as liberate bioactive peptides that are beneficial for heart and metabolic health. Recent research shows that microbial fermentation can remove protease inhibitors with up to 20% greater protein digestibility *in vitro* in pulse concentrates (93). Furthermore, enzymatic modification of protein structure is being investigated to introduce changes in the functional properties and decrease the allergenicity of proteins (94). The energy-intensive processing steps are necessary to guarantee that sustainable proteins are not only environmentally friendly but also nutritionally superior and bioavailable. The blending, fortification, and innovative processing of these improvement strategies will play a key role in realizing a circular bioeconomy and ensuring large-scale acceptance by consumers and protection of public health in future food systems (95). Nutritional characteristics and protein quality indicators of major sustainable protein sources are summarized in Table 1.

**Table 1:** Nutritional characteristics and protein quality indicators of major sustainable protein sources.

Protein source	PDCAAS/ DIAAS	Key amino acid profile (essential/limiting)	Digestibility (%)	Key micronutrients (Fe, Zn, B12, Ca, Omega-3)	Functional and health components	References
Soy (plant)	PDCAAS: 0.91–1.0	Complete EAA; limiting: methionine (Met)	95%−98%	Fe: 5.1 mg; Zn: 3.7 mg; Ca: 277 mg (per 100 g); B12: none	Isoflavones, saponins, dietary fiber	(240)
	DIAAS: 0.84–0.90					
Pea (plant)	PDCAAS: 0.67–0.78	Limiting: Met + cysteine (Cys)	88%−92%	Fe: 1.5 mg; Zn: 1.2 mg; Ca: 25 mg; omega-3: low	Fiber, Polyphenols	(241)
	DIAAS: 0.62–0.71					
Mycoprotein (fungi)	PDCAAS: 0.91–0.99	Complete EAA; high lysine/Leucine	85%−90%	Fe: 0.5 mg; Zn: 6.7 mg; B12: trace (unless fortified)	Beta-glucans, high dietary fiber (chitin/glucan)	(6)
	DIAAS: 0.85–0.92					
Insects (mealworm)	PDCAAS: 0.82–0.91	Limiting: Met + Cys or tryptophan (Trp)	78%−99%	Fe: 4.2 mg; Zn: 12.5 mg; B12: 0.47 μg; Ca: high	Chitin (fiber), bioactive peptides, lauric acid	(64)
	DIAAS: 0.72–0.88					
Spirulina (algae)	PDCAAS: 0.75–0.85	Complete EAA; Limiting: Met/Lys	81%−86%	Fe: 28.5 mg; Zn: 2.0 mg; B12: high (analog); omega-3: GLA	Phycocyanin, antioxidants, carotenoids	(242)
	DIAAS: 0.70–0.80					
Beef (animal Ref)	PDCAAS: 1.00	Complete EAA; no limiting AA	98%−100%	Fe: 2.6 mg (heme); Zn: 4.8 mg; B12: 2.6 μg; Ca: 18 mg	High bioavailability of minerals	(243)
	DIAAS: 1.00+					

## 5 Environmental sustainability and trade-off analysis

### 5.1 Environmental assessment frameworks

The shift toward sustainable food systems requires robust environmental assessment tools to evaluate the sustainability of alternative protein sources, including plant-based, insect-based, and cultured meats. Life cycle assessment (LCA) is the most commonly used scientific framework for this evaluation, generally employing a cradle-to-gate boundary that includes all production phases from the extraction of raw materials to the manufacture of the product (96). Modern LCA approaches and methods have developed to consider the particularities of new protein technologies by using modular eco-efficiency approaches and ex-ante assessment. Ex-ante LCA models are also important for cultured meat because they rely on primary data from pilot production facilities and computational projections to estimate the environmental consequences of future commercial-scale production before large-scale adoption and to evaluate their environmental impact (97). Moreover, the growing use of regionalized dynamic weighting methods to reflect geographical differences in land availability and policy contexts ensures that environmental burdens are framed within their respective ecosystems (98).

A critical component of these frameworks is carbon footprinting, which consistently demonstrates the potential of alternative proteins to achieve substantial reductions in GHG emissions compared to conventional livestock. Plant-based alternatives, such as chickpea-based burgers, can reduce GHG emissions by up to 92% relative to conventional beef (96). Similarly, cultured meat is projected to produce approximately 87% fewer GHG emissions than traditional beef production (99). However, the environmental performance of these technologies is highly sensitive to the energy mix used during processing; the use of renewable energy is essential for ensuring that cultured meat and highly processed plant-based proteins maintain a lower carbon profile than more efficient animal sources like poultry (100). Insect farming is also a very interesting low-carbon option, as the final environmental impact depends greatly on the technology employed for the insect protein powder (e.g., hot-pressing, oven-drying, etc.) (101). While the clear benefits of carbon reduction are evident, multi-impact assessment techniques are needed to control the inevitable environmental trade-offs that will be encountered in a comprehensive evaluation. Alternative proteins are generally more efficient in land and water use than livestock; however, they introduce new burdens, especially in terms of high energy use for bioreactor operations or intensive industrial processing (1). For instance, cultured meat uses much less land and water than traditional beef but is limited by the energy-intensive sterile environments and bioreactor systems required. Insect protein, however, has significant flexibility in feed choice, as waste-based feeds can have a lower overall effect than high-quality substrates (102), but may lead to lower production efficiency. Hence, researchers call for integrated indicators to take into account GHG emissions, water scarcity, energy requirements, and land use to prevent burden shifting to other resources when alternative proteins are used. This multi-dimensional analysis is crucial to guide policy and consumer decisions toward truly sustainable future food systems (101).

### 5.2 Greenhouse gas emissions

The GHG emissions resulting from livestock production are an important indicator of the sustainability of future food systems, and livestock production accounts for a considerable share of global warming, with methane, nitrous oxide, and carbon dioxide all being emitted. One of the main sources of these emissions is conventional animal agriculture, especially ruminant farming, with an estimated 8.01 times the CO_2_-equivalent (CO_2_-eq) emissions of equivalent plant-based meat substitutes from ground beef production (103). Even greater reductions can be made by replacing beef with microbial proteins obtained by geothermal energy, such as Spirulina, which can save around 100 kg CO_2_-eq per kg of protein produced (representing more than 99% of the CO_2_-eq emitted compared to beef) (104). When compared to alternative protein sources like pigs, which emit about 4.62 kg CO_2_-eq per kg of meat, alternative protein sources outperform pigs in terms of climate performance. Edible insects are highly sustainable, with efficient feed conversion ratios and much lower usage of fossil resources and climate-altering emissions than traditional pork and poultry production systems (105). The climate effects of cultured meat are more complicated since most of the carbon dioxide is due to energy use, unlike the carbon dioxide emitted from live animal production, which is associated with methane and nitrous oxide emissions. Although current calculations indicate that cultured meat may be nearly three times as efficient as chicken in converting crops into edible protein, its total “carbon footprint” during large-scale industrial production will vary significantly according to the energy mix used to produce this meat (100). The energy requirements for producing and maintaining the temperature of the bioreactor and the media used to grow the food could make cultured meat not much better than beef and pork and not competitive with the most optimistic sustainability targets for poultry if it relies on a traditional electricity grid that is heavily dependent on fossil fuels. However, if it is powered by renewable electricity sources, cultured meat is superior to both beef and pork and can be competitive with the most ambitious sustainability targets for poultry (97). In addition, since these alternate proteins are produced in closed biotechnological processes, they do not emit nitrogen pollution from manure and do not require land-intensive conventional grazing. Therefore, the GHG mitigation potential of sustainable proteins can be significant, but it will only be achieved when the transition to renewable energy infrastructures and the optimization of biotechnological processes are synchronized to ensure the removal of environmental impacts, not only from one impact category to another (104).

### 5.3 Land-use efficiency

The extensive use of land is one of the major environmental issues for traditional livestock production, especially beef, as large areas of land are needed for grazing and producing feed crops (105). The resources used in conventional meat systems are up to 70% higher than alternative protein systems, with beef having the highest land use per kg of protein produced (106). In contrast, alternative proteins such as plant-based proteins, insects, and cultured meat have the potential to provide significant efficiencies in land use for human nutrition. Plant protein is more efficient because it is a direct conversion of the crop to protein; optimizing plant protein diets using a 40:60 animal to plant protein ratio has been demonstrated to greatly reduce the amount of land necessary compared to high-meat diets (107). Insect protein uses land highly efficiently, orders of magnitude less than traditional livestock, per unit of protein. This efficiency is based on the fact that insects can be part of a circular economy by recycling nutrients from organic side-streams and agricultural wastes, thereby reducing the overall agricultural land area required to produce the feed (108). Likewise, cultivated meat offers a radical change in how land is used, with recent life cycle studies indicating it may be around three times as efficient at converting agricultural crops to meat as poultry, the most land-efficient conventional animal protein source. Cultivated meat eliminates the need for extensive grazing lands and does not require land applications as part of conventional waste management systems because it is created in vertically stackable, or contained, bioreactor systems (97).

However, the transition to these land-efficient alternatives involves complex trade-offs. Compared to traditional pastoral systems, the production of cultivated meat and highly processed plant-based analogs is much more energy-intensive, whereas land use is reduced. The benefit in terms of land use associated with these high-tech systems may be offset by a carbon footprint associated with the energy used in that system if it is not renewable (109). Moreover, sustainable protein production requires balancing land conservation with the sustainability of feedstocks and specialized medium formulations. Apart from the biodiversity benefits, the decrease in the demand for agricultural land offers an important chance to restore habitats and preserve natural ecosystems, critical to reducing the global extinction crisis. Land-use efficiency is a key part of the conversation about sustainable protein transitions, but it must be assessed against energy needs and comprehensive resource management to reach a truly regenerative and secure future food system (110).

### 5.4 Water footprint

Freshwater consumption is a key consideration for assessing the environmental sustainability and future food security of different protein sources, as the intensification of global food production has put incredible pressure on water resources. The amount of freshwater needed by conventional animal protein sources is significantly higher than that for alternative sources. For instance, producing 1 kg of protein from beef takes about 15,400 liters of water, reflecting the water used for intensive irrigation for feed crops and directly by animals. Plants, on the other hand, provide much greater water efficiency. A traditional beef burger can require up to 21 times the water resources of a vegetarian burger made from soy, beans, and rice (111). This difference indicates the possibility of a protein transition as a solution to reduce hydrological stress in water-deficit areas (112). These water footprints are usually divided into blue water (surface water/groundwater), green water (rainwater stored in the soil), and gray water (freshwater needed to dilute pollutants to ambient standards) (113). The green water footprint dominates in conventional livestock systems because of the large extent of pasture and fodder (often eight times the amount of land used for human food and nutrition), while the blue and gray water impacts are more important for local ecosystem health (114). Plant-based meat alternatives and hybrid products represent an option for a healthy compromise: hybrid burgers consist of 1.8 times less water than meat-only ones and have been shown to offer a viable alternative that reduces immediate environmental impact without requiring a complete dietary revolution. Edible insects are another potential alternative protein that also reduces environmental impacts. There are species known for their resilience in low-water conditions and ability to provide good nutritional value, such as crickets (115). Unlike conventional livestock systems that are major consumers of non-renewable water, insect and microbial proteins can be produced in a controlled environment that enables more precise water use and recycling. The trade-offs involve the energy-intensive nature of high-tech protein production processes, such as cultivated meat, where the large amount of cooling and processing water needed must be considered against the water savings in agriculture. Finally, diversification of protein sources to sustainable proteins is crucial to satisfy global demand and maintain the integrity of the hydrological cycle. These options offer a solution to balance improved nutrition with the need to safeguard limited water resources for the benefit of future generations (116).

### 5.5 Energy requirements

The shift from animal-based protein to alternative proteins requires a paradigm shift in the way energy is used, moving from the metabolic processes of animals to large-scale engineering systems. Other sustainable protein sources, such as cultured meat and insect protein, have many energy-intensity trade-offs, but plant-based protein typically has the least energy-intensive and lowest greenhouse gas emissions per protein unit (117). Beef production is traditionally very resource-intensive and strongly dependent on land and biological feed conversion. However, the production of cultivated meat is energy-intensive in its own right, requiring precise thermophilic conditions in bioreactors and energy-intensive biotechnological production of culture media (117). While one LCA estimates that a novel cultivated burger patty could cut cumulative energy demand by 39% compared to conventional beef (99), cellular agriculture could consume up to one-third of the projected global green energy capacity by 2050 if cellular beef were to be fully substituted. This transition effectively replaces large land and water consumption (which is achievable by more than 90% in cultivated systems) with a greater dependence on the decarbonization of the electrical grid (118). The energy requirement of insect farming is different, where thermal management is the main challenge. The energy use in the production of yellow mealworms (*Tenebrio molitor*) has been estimated at about 213.66 MJ-eq per kg protein, with on-farm heating representing up to 65 percent of the environmental impact. This energy demand is considerable but is frequently compensated for by the higher feed conversion ratios and lower water use of insects compared to broiler chickens or swine (119). Likewise, in PF and microbial protein production, high energy consumption is needed for agitation, aeration, and sterilization in large-scale fermenters. The viability of such systems is not guaranteed but rather depends on the energy paradigm of the respective production area (100). Cultivated meat generally has lower GHG emissions than beef but varies widely and is sensitive to the amount of renewables in the electricity grid when compared to pork or poultry. In conclusion, the energy demand of future food systems is a key trade-off: mitigating land-use change and biodiversity loss through intensified industrial production can only be truly sustainable if combined with a sharp shift to renewable energy infrastructures (1).

### 5.6 Biodiversity and ecosystem impacts

The livestock industry is still the primary cause of biodiversity loss worldwide, largely because of its large land footprint and resulting habitat destruction. Traditional animal agriculture utilizes almost 80% of global agricultural land, both in pasture and for cropland used for animal feed, but delivers only a small portion of the world's food calories (120). Such inefficiency contributes to the loss of high-value ecosystems, especially forests and grasslands, to agricultural monocultures, which fragment habitats and drive many species to extinction. The use of sustainable protein alternatives, such as plant-based analogs, edible insects, cultured meat, and precision fermentation, offers a paradigm-changing pathway to reducing these harmful environmental effects from protein production. Replacing 50% of conventional meat and milk with plant-based alternatives is enough to stop the net loss of forests and natural land by 2050. This transition would help maintain current biodiversity hotspots while also enabling ecological restoration on a massive scale; areas where livestock are excluded could help achieve up to 25% of the restoration goals called for in the Kunming-Montreal Global Biodiversity Framework (121). In addition to land conservation, alternative proteins reduce other ecosystem stressors like nutrient pollution. Conventional livestock farming is a key driver of nutrient pollution (nitrogen, phosphorus) in freshwater and marine environments, leading to eutrophication, which is devastating to aquatic biodiversity. Plant-based systems also need nutrient inputs, but the possibility of adding nitrogen-fixing crops lessens the chemical footprint on the environment (122). In addition, insects possess remarkable feed conversion ratios and can be raised on organic waste streams to not only reduce land footprints but also virgin feed crops (123). However, the shift to alternative proteins is not a simple ecological equation without its own complexities and trade-offs. A transition to plant-based diets could shift environmental pressures and create a greater need for water and pollination services in certain areas, potentially compromising local ecosystems unless managed sustainably. In addition, the sustainability of cultured meat and PF depends on the energy mix, as fossil fuel production will have indirect effects on biodiversity due to climate impacts. Furthermore, harvesting wild insects in some areas carries the risk of over-exploitation, potentially disrupting local food webs (124). To reach the maximum biodiversity value of sustainable proteins, integrated land-use policies need to be developed, in which spared land is used with the positive aim of restoring it instead of moving it to other intensive industrial applications (125).

### 5.7 Resource circularity and waste reduction

The shift to sustainable food systems requires moving from linear take-make-dispose to bio-economies that focus on resource efficiency and waste reduction. Resource circularity in protein production involves the strategic reconversion of by-products and side-streams with high nutrient value into human food or animal feed. Cereal processing wastes, oilseed cakes, legume husks, and other agro-industrial residues are a large untapped source of functional proteins (126). For instance, brewer's spent yeast is a major by-product of the brewing industry and can be processed into high-quality protein isolates for human consumption via innovative membrane filtration and enzymatic processes. The use of green extraction technologies, including ultrasound-assisted and microwave-assisted extraction, further improves the sustainability of these processes by minimizing the use of chemical solvents and maintaining the bioactivity of the extracted proteins, including antioxidant and antihypertensive peptides (127). Another significant aspect of resource circularity is insect-based bioconversion, with species such as the black soldier fly (*Hermetia illucens*). These insects are capable of efficiently converting organic food waste and agricultural wastes into rich biomass, lipids, and essential amino acids (128). Besides helping divert waste from landfills and reducing methane emissions, this also offers a sustainable feed alternative to finite feed sources such as soybean meal and fishmeal in aquaculture and animal feeds. Likewise, the marine industry is moving toward zero-waste biorefinery processes to maximize the value of the 50%−60% seafood waste generated during processing. The use of green refining methods allows for the recovery of proteins and nutraceuticals from viscera and bones and for nutrient-rich effluents to be re-used as growing media for microalgae, thus forming closed-loop systems that reduce environmental discharge (129). Microbial protein production using precision fermentation is another example of circularity, where hydrolysates of industrial residues serve as feedstocks. Ultimately, these biotechnological innovations enable the production of non-animal proteins on a much larger scale and with significantly less environmental impact than conventional animal production. However, there are some caveats when it comes to incorporating circular principles. Some recovery methods have high energy requirements, while the raw waste materials are not always of high quality, creating economic and operational barriers to widespread implementation (130). To attain a truly circular approach, a multidisciplinary strategy is needed to optimize nutrient recovery systems and to guarantee the safety and scalability of upcycled ingredients. The global protein industry can reduce risk in production chains, strengthen food security, and approach the United Nations Sustainable Development Goals through the valorization of biomass losses (130). In conclusion, the implementation of these systems is heavily reliant on the integration of technological advancements with regulatory backing and consumer acceptance of upcycled protein sources, as well as achieving environmental benefits that support nutrition.

### 5.8 Environmental trade-offs among protein sources

**Figure 2:**
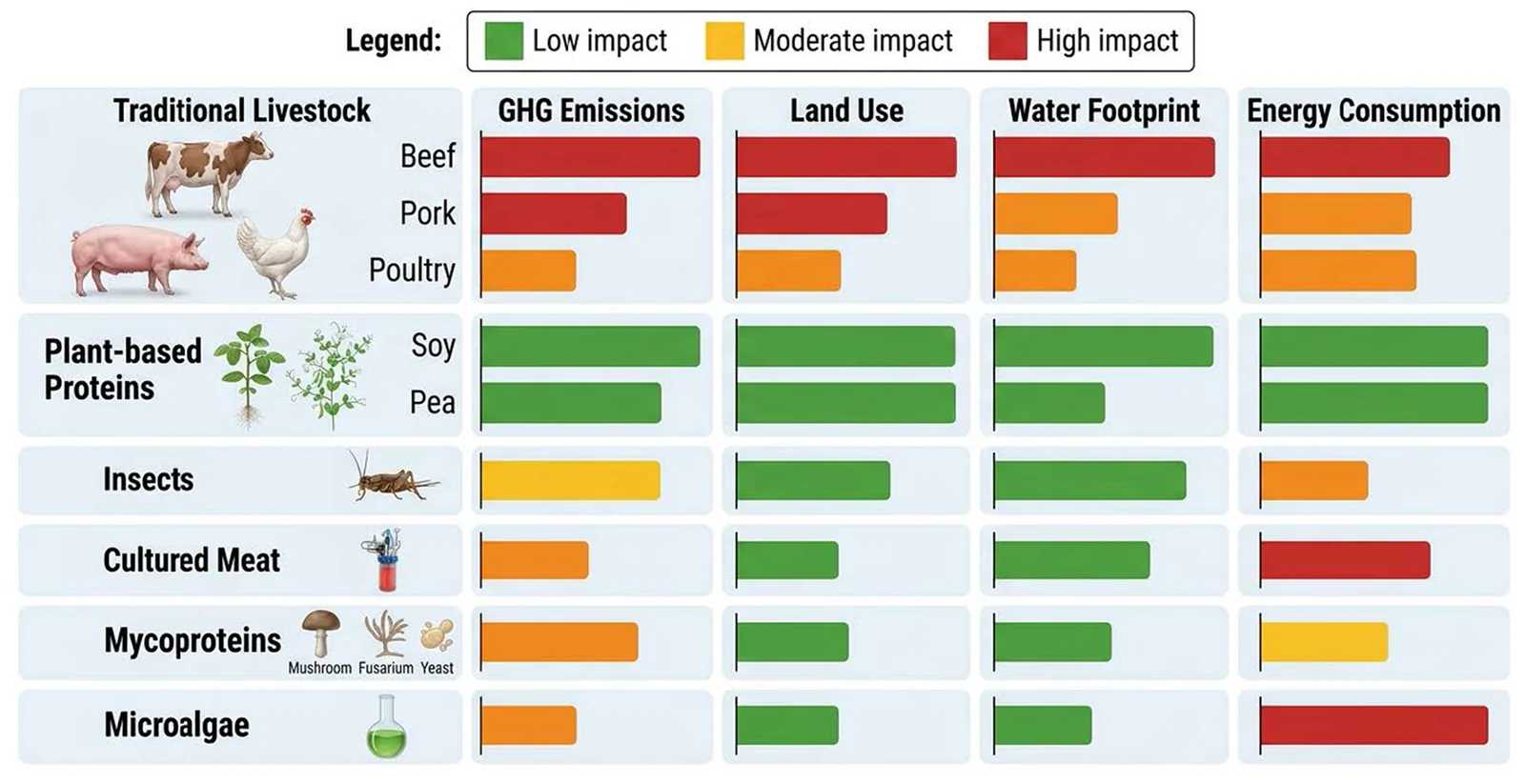
Environmental trade-offs across major protein production systems. The impact categories (low, moderate, high) shown in this figure and Table 2 represent qualitative bands rather than a single continuous, directly comparable metric. Environmental impact metrics (per kg protein) are shown as min–max ranges (values/references in Table 2): GHG emissions (kg CO_2_-eq), land use (m^2^), water footprint (L), energy demand (MJ). Plant- and novel alternative proteins exhibit reduced GHG, land, and water footprints relative to conventional meat; however, high energy inputs partially negate these gains for cultured meat and mycoprotein. Wide data ranges reflect variability from production methods, energy sources, and geographic conditions. Created with BioRender.com.

The environmental impact of protein production is also highly dependent on the protein source, thus leading to intricate frameworks of trade-offs between climate impact and resources used (Figure 2). Conventional beef production is always the most resource-intensive source, and life cycle assessments show it generates around eight times the amount of global warming compared to plant-based options, and nearly three times more land use (103). These burdens are further intensified when accounting for land-use changes such as deforestation for pasture (104). However, in comparison, production of poultry and swine tends to have lower GHG emission intensities and lower land use than ruminants, but still has a higher environmental profile than low-processed plant protein such as legumes (131). Plant-based meat analogs (PBMAs) fall somewhere in between: they are 62% lower in GWP than beef burgers, but their high production demand and complexity can lead to hidden ecological costs due to their manufacturing process and long supply chain. PBMAs are very efficient in land and water use, but can require intensive processing to achieve sensory properties that are in line with those of animal meat, which can lead to higher energy use than whole-plant foods (132).

Likewise, insect proteins are becoming popular for their potential to produce quality protein by utilizing organic waste with less need for land and water, and with lower energy requirements for climate control in rearing systems. However, their environmental benefits are sensitive to the feed substrate used and the energy needed to control the climate inside the rearing facilities. Technological advancements like cellular agriculture and microalgae cultivation offer an even greater set of trade-offs. The potential for cultured meat to limit land and water usage is more than 90% less than beef, but this reduction is very dependent on the energy source used for bioreactors (99). The energy consumed by cultivating cells is substantial, and if no transition to renewable energy is made, the electricity consumed in cellular production will be as much as or more than that of intensive poultry production (100). The integration of microalgae (e.g., *Arthrospiral platensis* and *Spirulina*) into geothermal biorefining systems with low carbon footprints and low land requirements is challenging due to economic constraints and infrastructure requirements for economically viable large-scale deployments. Moreover, the shift to these high-tech systems calls for more critical materials, potentially causing bottlenecks in the global transition toward sustainable diets (118). In conclusion, alternative proteins can provide some promising solutions to reduce the impact of animal production on the environment, but the full potential for sustainability will depend on a consideration of the various trade-offs in relation to nutrient bioavailability and the energy needed to produce proteins (132). Comparative environmental footprints of conventional and sustainable protein sources are summarized in Table 2.

**Table 2:** Comparative environmental footprints of conventional and sustainable protein sources.

Protein source	GHG emissions (kg CO_2_-eq/kg protein)	Land use (m^2^/kg protein)	Water footprint (L/kg protein)	Energy requirements (MJ/kg protein)	References
Conventional sources					
Beef	250–1,000	150–400	15,000–20,000	30–50	(99)
Pork	50–100	40–60	4,000–6,000	25–45	(244)
Chicken	30–60	30–50	3,000–4,500	20–35	(245)
Sustainable alternatives					
Pulses/legumes	1–5	5–15	100–500	5–10	(246)
Soy protein	1–3	5–10	150–300	8–12	(117)
Insect protein	2–15	1–15	10–200	20–100	(246)
Cultured meat	5–25	1–5	100–500	100–250	(118)
Mycoprotein	5–10	2–5	500–1,000	50–150	(245)

## 6 Sustainable proteins within circular food systems

### 6.1 Circular economy principles in protein production

The adoption of circular economy concepts in protein production represents a transformative shift from linear take-make-waste systems to systems characterized by minimal environmental leakage and high resource efficiency. An important aspect of this transition is the valorization of agri-food side-streams, which are produced in large quantities during harvesting and processing but are underutilized. The food industry can help enhance food security and reduce price volatility by creating valuable protein ingredients from these low-value materials (126). The co-products of renewable ethanol fermentation are becoming an attractive source for high-value protein production, offering an alternative to conventional feedstocks that can be grown with minimal impact. Similarly, novel protein recovery from crop processing waste, including genome-edited potato protein, has shown remarkable potential for mitigating terrestrial acidification and land use compared to conventional agriculture (132). These circular strategies contribute to international sustainability targets, such as zero-waste systems and minimizing the use of virgin materials. Biological conversion agents, such as insects and microorganisms, play a vital role in these circular systems by serving as decentralized bioreactors that convert nutrients into human consumables (133). Filamentous fungi can be fermented on many agro-industrial residues to obtain valuable mycoproteins that are rich in EAAs and minerals. The use of microbial platforms is highly efficient, requiring only a fraction of the land and water usage compared to livestock. Moreover, SCPs can be synthesized from CO_2_ captured from manufacturing processes and nutrients recovered from anaerobic digestion digestate, thus transforming waste emissions into nutrient-rich biomass (134). Meanwhile, insect-based biotransformation using insects such as the black soldier fly and yellow mealworm provides a strong framework for transforming organic food waste into valuable feed and food ingredients. This mechanism makes the food system more resilient for local communities, thereby decreasing the carbon footprint associated with long-distance transport (135). One of the basic goals of circular protein systems is the closure of nutrient cycles, especially for N and P, which are often lost with conventional disposal. In circular systems, these nutrients are incorporated into microbial or insect biomass and are not discharged into ecosystems, thereby lessening the risk of eutrophication and other environmental imbalances. The effectiveness and efficiency of these systems will rely on strategic planning of feedstock availability and matching technological capability with regulatory safety guidelines. LCA coupled with geographical mapping can be used to optimize supply chains, making circular protein production economically viable and environmentally beneficial. These technologies will play a key role in future sustainable food systems, providing a scalable answer to the global protein challenge and contributing to planetary health, once they become mature and approved for use (136).

### 6.2 Agricultural and food by-product valorization

The shift toward circular food systems requires strategic valorization of agricultural and food processing by-products, which turns what would have been considered waste into high-value, sustainable protein sources. Oilseed meals such as sunflower, flaxseed, brewery spent grains, and even abattoir residues like dried blood-rumen mixtures are enormous untapped stocks of nutrients (137). These proteins have been extracted and processed using sophisticated biotech and mechanical methods. Mechanical stirring, ultrasound-assisted extraction, and CO_2_-based methods are currently used to extract protein flours from sunflower meal for human use (138). At the same time, agri-food residues can be used as substrates for filamentous fungi and other microorganisms by means of microbial bioconversion processes, thus providing nutrient-rich biomass. AI-driven systems further optimize these processes to generate proteins with high scalability and traceability, adhering to industrial standards (24). Proteins from by-product valorization may be nutritionally superior to conventional protein sources in terms of quality and density. Fungal, yeast, and algae single-cell proteins contain protein levels of 30% to 80%, often surpassing the protein levels of conventional meat and soy products. Mycoproteins from fungal fermentation have a complete amino acid profile (essential amino acids) and include high levels of minerals like phosphorus, zinc, and iron, as well as beneficial dietary fibers (139). From an animal nutrition perspective, dried blood-rumen content mixtures from slaughterhouse side streams can be used to supply up to 80% crude protein, which in turn can enhance animal growth performance and minimize environmental impacts associated with waste disposal. The consequences of this valorization are far-reaching for the environment and economy. The incorporation of these side streams into the production cycle allows for the utilization of less resource-intensive crops, such as soybean, and fishmeal, which can be linked to deforestation and overfishing (128). For instance, the carbon footprint of mycoprotein production from agricultural waste can be more than 10 times more favorable than the carbon footprint of beef production. From an economic perspective, the use of organic residues as substrates could reduce protein production costs by 35%−75%, contributing to the development of resilient and cost-effective food systems (128).

Nevertheless, there are some barriers to adoption. Scaling up production demands significant investment in cost-effective mass-rearing and processing technologies. In addition, creating uniform safety regulations and gaining consumer acceptance of “waste-derived” foods are important challenges to address. Improving sensory properties and guaranteeing clear regulatory approvals will be critical to establishing credibility with the public and for the successful market launch of these innovations in protein recycling (139).

### 6.3 Nutrient recovery and recycling

To achieve circular food systems, the paradigm needs to change from linear take-make-dispose to closed-loop systems where nutrient recovery and recycling become paramount aspects of sustainable protein production. Industrial food systems are responsible for large losses of reactive nitrogen and phosphorus to the environment, much of which is lost into wastewater, manure, or food processing by-products, contributing to widespread eutrophication and soil acidification (140). These nutrients can be recovered from liquid waste streams using advanced recovery methods like partition–release–recover (PRR) technologies, chemical precipitation, and advanced membrane filtration, and then put back into the production cycle (141). Among the most promising uses of these recovered nutrients is the growth of microbial proteins or SCP, which include a variety of bacteria, fungi, and microalgae. These microorganisms can convert organic carbon and inorganic nitrogen into high-density protein biomass, relying largely on non-agricultural land and freshwater resources. Ammonia and carbon dioxide can, for example, be used as the main feedstocks for methanotrophic and hydrogenotrophic bacteria to produce nutritionally rich animal feed, an alternative to resource-heavy soybean meal and fishmeal. The incorporation of Power-to-X technologies further supports this process by producing the hydrogen needed for protein production from renewable energy, thus eliminating the need for fossil fuels (142). Environmentally, closed-loop microbial protein synthesis presents significant advantages, such as a global warming potential that is significantly less than conventional wastewater treatment designs. Furthermore, recycling phosphorus helps to lower the global consumption of limited phosphate rock resources and synthetic fertilizers, which also pose high energy costs and violate planetary boundaries (141). Nutritionally, these recovered proteins frequently exhibit amino acid profiles akin to conventional high-quality protein sources, and some microalgae and fungi species are able to provide extra bioactives and beneficial fibers, as well as minerals such as phosphorus and zinc. Even though the technology is feasible and green, there are still several structural challenges to overcome for wider adoption. Food safety regulations are often strict and consequently restrict the use of proteins from waste for human consumption, limiting them to animal feed, due to the possible risk of heavy metals being concentrated or pathogens being passed from the waste stream into the final product (143). Moreover, the economic feasibility of these systems is still dependent on energy prices, technological readiness, and market rivalry with global commodity goods. However, the solutions to these challenges lie in continued investment in multi-faceted techno-economic evaluations and in creating a regulatory structure that sees recovered nutrients as valuable resources instead of waste. Finally, nutrient recycling in a circular food system will be crucial to ensure long-term global food security without exceeding the safe operating space of our planet (144).

### 6.4 Biorefineries and integrated production systems

The multi-product biorefinery concept marks a paradigm shift from mono-product biorefineries to systems that enhance the value of raw material usage in circular food systems (145). These facilities are vital for the production of high-value proteins from diverse biomass, in addition to producing biofuels, chemicals, and materials, thereby helping to separate the food chain from resource-intensive practices. Agricultural wastes are being increasingly used to generate protein-rich biomass for animal feed and polyhydroxyalkanoate (PHA) bioplastics for sustainable packaging in dual-stream biorefineries. Likewise, green biorefinery platforms use forage crops such as clover, ryegrass, and alfalfa to produce leaf protein concentrate (LPC), bio-crude, bio-gas, and bio-fertilizers (146). This integration goes beyond using green biomass; grain-based bioethanol plants have been re-engineered to generate high-quality protein feed as a by-product, which has a positive impact on regional protein security and decreases the need for imported soybean meal (147). These integrated systems are highly efficient, depending on the complexity of the mechanical, chemical, and biological technologies involved in their design and operation. Mechanical fractionation first separates fiber-rich solids from nutrient-dense juices, which are then processed via heat treatment and centrifugation to isolate protein fractions (148). The side-stream carbon and nitrogen are further valorized by enzymatic hydrolysis and microbial fermentation for the production of single-cell proteins or bioplastics. Advanced integration is illustrated by emerging “power-to-protein” technologies, which use renewable energy and waste nutrients to produce proteins for feed formulations with minimal environmental impact (149). Integrated biorefineries provide significant advantages in terms of resource efficiency and climate protection from a sustainability point of view. LCA studies have shown that these platforms can cut GHG emissions by up to 43% compared to traditional fossil-fuel-based production due to the valorization of side streams like press cake and brown juice in bioenergy. Moreover, these systems achieve outstanding rates of carbon and nitrogen recovery, critical for planetary boundaries (146). The co-production model is also more economically resilient, leading to lower protein production costs, some of which are estimated at as low as $1 per kg of dry biomass. The move to integrated production systems entails certain technological and operational compromises. Multiple unit operations (hydrothermal liquefaction, anaerobic digestion, precision fermentation) make their management highly complex. However, energy intensity is still an issue, especially in downstream separation and drying processes, and more innovations are needed in membrane technology and heat recovery. Secondly, there is a need to consider land use, as the growth of dedicated protein crops could compete with current food or forage crops. However, sustainable protein production worldwide requires integrated biorefineries to overcome these hurdles (150).

### 6.5 Waste-to-protein technologies

The shift toward a circular bioeconomy requires the incorporation of waste-to-protein technologies that can help valorize organic side-streams that would otherwise be a source of environmental pollution (151). These technologies are based on the use of biological agents such as insects, fungi, bacteria, and algae to recover nitrogen and carbon from agricultural, industrial, and municipal waste (152). One example of this shift is the emergence of insect farming, specifically with the black soldier fly (*Hermetia illucens*). If insects are raised on organic wastes, they can be a source of high-quality protein biomass with reduced environmental impacts compared with conventional livestock. For example, 1 kg of protein from insects requires about 1.5 m^2^ of land and 0.1 m^3^ of water, and emits 1 kg CO_2_ eq. in GHGs. In addition to being environmentally efficient, insects also have high feed conversion ratios and can be part of a circular economy, where low-value agri-food waste is converted into high-value feed or food components (153). In addition to insect-based systems, microbial protein production is a highly scalable option for the upcycling of diverse feedstocks. SCP is the mass cultivation of microorganisms, such as yeast, bacteria, or microalgae, on substrates such as pineapple waste, lignocellulosic biomass, or digestate from anaerobic digestion (AD) with the addition of CO_2_ from the upgrading of biogas (154). Recent developments have shown that such use of real nutrient-rich digestate materials can quadruple the biomass production rate over synthetic options, with doubling rates as short as 15 h. Although SCP production is energy-intensive, with approximately 10 kWh/kg energy consumption, the limited land requirement (0.5 m^2^) and the possibility to separate protein production from arable land use makes it an important tool in the world's food security. Likewise, the rapid growth rate of filamentous fungi, such as *Fusarium* and *Rhizopus*, has been used for the production of mycoprotein, which is the edible protein obtained from starch-rich food processing by-products. These fungal biorefineries enable the recovery of nutrients from side-streams while generating fungal biomass with higher protein and fiber content as well as diverse functional properties for use in nutraceuticals and biodegradable packaging (155). The broader upcycling of food industry by-products requires a two-step process, including the direct extraction of proteins from solid and liquid food industry wastes (chemical solubilization and membrane filtration), as well as the conversion of the nutrient residue into SCP. This combination is the most efficient use of raw materials, turning what is a liability into an asset. However, there are serious technological and socio-economic challenges in the way. These include problems of process standardization from the food safety point of view, the processing energy demand of some microbial systems, and better consumer acceptance of waste proteins. To unlock the potential of waste-to-protein technologies, addressing these bottlenecks by optimizing processes with artificial intelligence and developing appropriate regulatory frameworks will be key to achieving a resilient and circular global food system (143).

### 6.6 Circular bioeconomy opportunities

**Figure 3:**
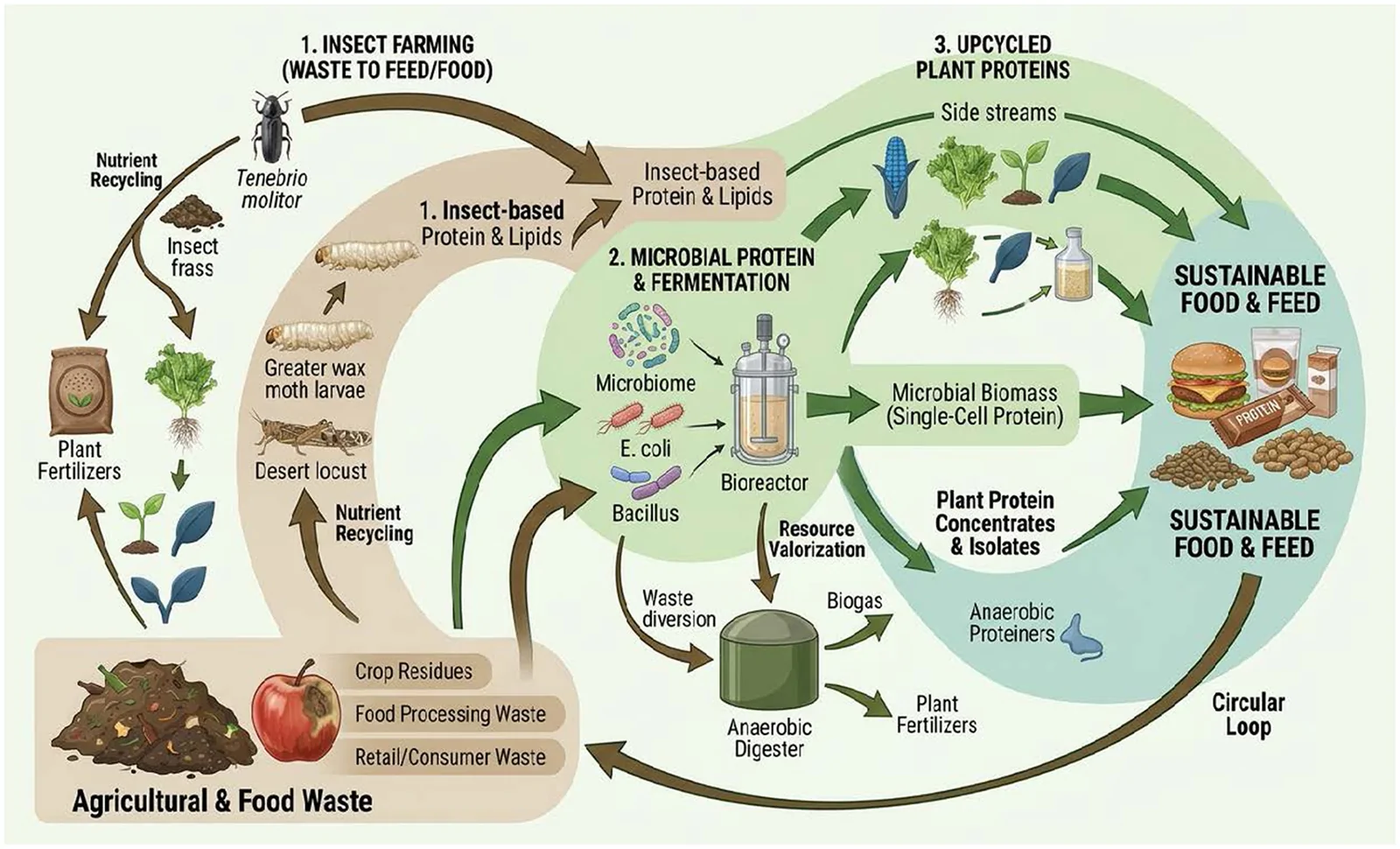
Circular food system framework integrating sustainable protein production and resource valorization. Created with BioRender.com.

To transition to a circular bioeconomy, transformations are needed, shifting protein production from linear take-make-waste systems toward closed-loop systems. One key potential lies in the transformation of a wide variety of organic waste streams—from agricultural crops to industrial side-streams and urban food waste—into high-value protein biomass (156). The microbial protein (MP) fermentation process can use low-cost substrates such as food waste or even CO_2_ and could help satisfy a large fraction of the world's protein needs, avoiding disposal in landfills (157). Similarly, the cultivation of black soldier fly larvae is a powerful resource recovery mechanism because they are polyphagous and can feed on a variety of food waste from markets and catering establishments. This waste can then be processed into feed for aquaculture and livestock production, providing a nutrient-rich alternative to soybeans and fish meal. This process reduces the environmental footprint associated with waste disposal and generates a beneficial product that can be used as an alternative protein source, replacing resource-intensive options like fishmeal or soybean meal (158). In addition to waste reduction, these circular models offer significant environmental advantages, such as massive decreases in land and water use compared to conventional animal-based farming. Fungal, bacterial, and algal SCP frequently contain protein levels ranging from 30 to 80%, sometimes higher than that of meat or legumes (Figure 3). These proteins could be integrated into the regional value chains of Farm to Institution to Farm (FITF) models, which can help create economic resilience for local communities by bringing food-service waste to insect or microbial farms and thus ensuring a continuous supply of sustainable protein (159). Economically, waste substrates could decrease the production cost of SCP by 35 to 75 percent compared with conventional substrates, due to their being free or low-cost. Additionally, co-products of these systems (insect frass or fermentation digestate) can be incorporated into the soil as organic fertilizers, closing the nutrient cycle and improving soil health (157). Technical and regulatory challenges must be addressed to fully realize the opportunities of a circular bioeconomy. Variability in waste feedstock quality, energy requirements for industrial fermentation, and standardized safety procedures regarding waste-derived proteins present significant challenges, which can be overcome with innovation in bioprocessing and infrastructure (159). Advanced pre-treatment processes like hydrothermal or microbial hydrolysis can be utilized to achieve more consistent production from diverse waste streams. The incorporation of sustainable proteins into a circular bioeconomy provides a scalable, environmentally friendly approach to the long-term provision of food security (156).

## 7 Consumer acceptance and market adoption

### 7.1 Current market trends

The global protein ecosystem is in a state of change, with the food sector anticipating a 70% rise in meat demand by 2050—a demand that traditional livestock production systems are not well-positioned to sustainably fulfill. In this dynamic market, plant-based meat analogs are well-positioned, having built a production infrastructure and achieved a degree of commercialization that has enabled them to gain traction in the mainstream food service and retail sectors (160). Current trends (2024–2026) suggest that consumers are becoming more aware of environmental issues such as greenhouse gas emissions and land use, but their purchasing decisions are most likely driven by the personal health, nutritional quality, and sensory experience of the product (161).

This individualistic wellbeing approach has shifted the focus of manufacturers to “cleaning up” labels and producing foods with less intensive processing that maintain the same sensory characteristics and protein bioavailability as animal-derived foods. Emerging proteins like cell-based (cultured) meat and insect-based proteins face a more intricate path, whereas plant-based proteins are relatively established. Although cultured meat has the potential to reduce environmental trade-offs, it is still limited by high production costs and the technical difficulties of large-scale bioreactor production (162). Likewise, insect proteins face high disgust barriers and cultural resistance in Western markets, where despite their high efficiency, they are still confined to niche applications, such as animal feeds. International safety and labeling standards vary, and regulatory frameworks remain a major barrier for these new sources to come to market (160). One of the most important changes in the latest market data is the rising number of people moving toward flexitarianism—individuals looking to cut down on animal protein rather than eliminate it entirely. This is leading to the development of hybrid products that contain both animal and plant protein, which will serve as a stepping stone to achieve consumer acceptance (163). However, the most important hurdle to the wider adoption of all categories of alternative protein is price parity. As of 2026, the industry is more interested in technological innovations for reducing costs and in strategic communication policies to overcome consumer skepticism. Clear labeling and public education are being used to help shift perceptions toward a more sustainable food system, as a reliance on a single source of protein will likely not sustain a global food supply over time. Finally, the market is turning from a black-and-white definition of quality, which prioritized sustainability, to a more nuanced one where taste, price, and health are ultimately the deciding factors in consumer choice and sustained market growth (162).

### 7.2 Drivers of consumer acceptance

Consumer acceptance is key to the protein transition, and the shift toward sustainable food systems inherently depends on large-scale dietary transformation. Various alternative proteins can be developed using technology, but their market success is most influenced by a complex set of motivational drivers. One of the key drivers of this transformation is health awareness, with the growing perception of sustainable proteins, like plant-based and microbial proteins, as better options than typical animal-based ones (164). This perception is motivated by the need to reduce saturated fat, boost fiber, and avoid chronic diseases associated with high intakes of red and processed meat. In the South Indian region, nutritional value is a non-negotiable attribute, where the healthiness of the product is one of the most critical factors influencing positive attitudes and intended adoption. In addition to health motivation, ecological awareness has become a strong motivational factor in recent years, particularly for younger and better-educated target groups who are increasingly considering the ecological aspects of their purchases (165). The necessity of sustainable proteins for planetary health has also been resonating in public conversation, given the important role that livestock play in greenhouse gas emissions, land use, water use, etc. Preliminary studies show that people with a strong commitment to environmental issues are more apt to embrace new proteins and are often willing to pay more for sustainable products with a certified sustainability seal (10). This green motivation is often linked with a greater sense of civic responsibility and a desire to contribute to climate change through their diet. Furthermore, ethical issues and animal welfare concerns impact consumer preference. Both vegetarians, vegans, and flexitarians have considered the moral outcomes of industrial animal farming and have wanted to reduce animal suffering. Cultured meat is also emerging as an ethical protein option, where the familiar sensory experiences of consuming animal protein can be delivered with a reduced animal welfare cost (164). This change toward compassionate consumption is the result of an increasing connection between food choice and personal identity, and ethical consistency is a sign of a sustainable lifestyle. The influence of these drivers may differ widely among cultures, with animal welfare being a major driver in European and North American markets, whereas religious or traditional ethical considerations may be dominant in other markets (164). In conclusion, although health, environment, and ethics offer a motivational base, the widespread adoption of sustainable proteins in food systems will need to overcome remaining issues of sensory appeal and cost to ensure that these motivations drive behavioral change (164).

### 7.3 Barriers to adoption

**Figure 4:**
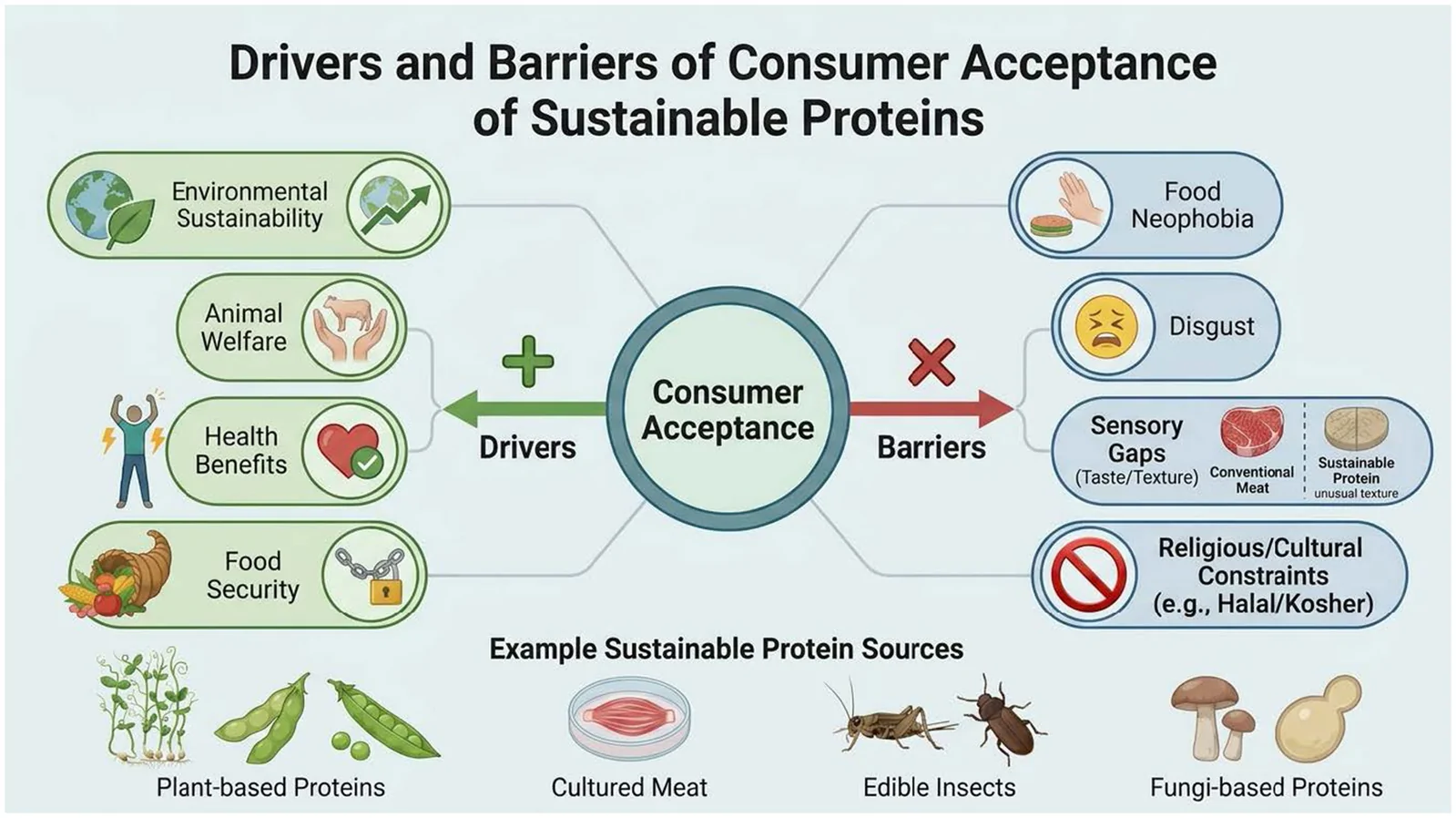
Drivers and barriers influencing consumer acceptance of sustainable proteins. This figure illustrates that factors influencing consumer acceptance function as either drivers or barriers depending on contextual conditions, individual differences, and cultural background. Individual-level factors (e.g., food neophobia, health motivations, and moral values), product-related characteristics (e.g., perceived naturalness, sensory attributes, and price), and socio-cultural influences (e.g., social norms, cultural food traditions, and institutional trust) interact bidirectionally to shape consumer acceptance. Created with BioRender.com.

The widespread adoption of sustainable proteins is critical for the resilience of future food systems, yet it remains hampered by a complex interplay of psychological and sensory barriers. The two biggest psychological barriers are food neophobia and food technology neophobia, which lead to a strong reluctance to try new protein sources like plant-based meat analogs, cell-cultured tissues, and edible insects. Such neophobic attitudes cause consumers to perceive products as having higher risks and lower benefits, even when the products offer real nutritional or environmental advantages (Figure 4) (166). In many Western societies, there is a particular disgust factor toward insect-based proteins, as they are considered culturally inappropriate or unhygienic to eat. The disgust response is an instinctive emotional reaction that is difficult to change, even with scientific data on sustainability or protein quality (167). The psychological mechanisms underlying disgust-based rejection of novel proteins are increasingly understood through the lens of moral foundations and purity concerns, rather than disgust alone as an isolated sensory reaction. Individual differences in aversion to tampering with nature predict discomfort with technologically produced foods such as cultured meat and, counterintuitively, have also been found to positively predict perceived benefits and willingness to consume cultivated meat once framing and nomenclature address underlying concerns, highlighting the complexity of this mechanism (168–170). Similarly, disgust sensitivity is closely tied to broader moral intuitions concerning purity and contamination, which can shape attitudes toward novel or technologically mediated foods independently of objective risk information (171). Incorporating these psychological and moral foundations frameworks into future consumer acceptance research on sustainable proteins would help explain individual and cultural variation in resistance beyond simple disgust ratings.

In addition to psychological resistance, the sensory characteristics (taste, texture, and smell) of these alternatives are also crucial to their market acceptance. Consumers often have negative sensory expectations of novel proteins, anticipating them to be less tasty and less pleasant to eat than traditional meat. For instance, insect-based products are sometimes described as gritty, while some plant-based products are described as beany or mushy (172). These sensory barriers are particularly troublesome because they make the shift from trial to regular, habitual consumption very difficult. Additionally, discomfort about the ontological nature of high-tech proteins, such as cell-cultured meat or heavily processed plant proteins, is another contributing factor (173).

Many consumers equate health and safety with naturalness and are therefore less likely to accept foods made in bioreactors or intensive chemical processes, considering them artificial or technological (173). It should be noted, however, that perceived naturalness functions as a distinct driver of consumer acceptance in its own right and is not simply a proxy or heuristic for health or safety; in some contexts, naturalness preferences are unrelated to, or even inversely related to, perceived healthiness or safety, particularly for preventative as opposed to curative health claims. This distinction matters because interventions addressing safety concerns alone may not resolve naturalness-based resistance to sustainable proteins (174).

This skepticism is frequently reinforced by a lack of transparency in the production process, resulting in a black box effect where consumers feel cut off from the origin of their food and its technological production process. These barriers can be overcome by adopting strategies that make these proteins more common in the current food system. Novel proteins can be transformed into familiar ones, such as by including insect powders in pasta or crackers, and research indicates this can reduce neophobic barriers (175). However, blending novel proteins into familiar foods is not uniformly beneficial: because blending changes the perceived composition and processing history of a food, it can also invoke essentialist or purity-based objections and provoke backlash rather than acceptance, particularly when consumers become aware that a familiar food has been altered with an unfamiliar or lab-derived ingredient (176). Consumer judgment also weighs added ingredients more heavily than equivalent removals (an “additivity dominance” effect), which can heighten sensitivity to blended formulations. Strategies relying on blending should therefore be evaluated for the risk of triggering purity concerns alongside their potential to reduce neophobia (177).

Furthermore, repeated exposure and social normalization are important to change consumer views from disgusting to socially desirable. Ultimately, sustainable proteins that are to be adopted in the mass market must not only be environmentally successful and nutritious, but must also break negative sensory stereotypes by providing a convenient, familiar, and delightful eating experience that is comparable to or better than the perception of traditional animal products (178).

### 7.4 Role of sensory quality

Sensory properties of sustainable proteins, ranging from taste and texture to appearance and aroma, are the most important drivers of consumer acceptance and the main obstacles to long-term dietary change. Although environmental attributes and moral values may trigger initial purchase interest in alternative proteins, the decision to buy again is virtually determined by the “hedonic” experience of eating them (173). PBMA continue to represent a significant technological hurdle in reaching “sensory parity” with animal-based products. The lack of juiciness, elasticity, and the presence of off-flavors are common consumer concerns, and are the primary reasons they are discouraged from eating them regularly (179). These off-aromas are generally inherent in the protein sources (soy or pea) and are difficult to mask using traditional strategies or to achieve the fibrous and chewy character of muscle tissue using basic processing methods (180). One of the sensory problems with cultured meat is recreating the complex architecture of whole muscle cuts. In addition to the cultured cells themselves, the distribution of intramuscular fat and the Maillard reaction products, which are characteristic of grilled beef or pork, are critical to meeting the expectations of flexitarian customers (181). Likewise, edible insects present a distinct sensory challenge: the cultural “disgust” factor. Consumers are more willing to eat insect flours when these are processed into an invisible flour, but for broad acceptance, insect-based foods need to be used by consumers as an ingredient for a desirable food product with its own appealing taste profile, typically referred to as nutty or umami (182). The interplay between sensory quality and nutritional perception also creates a complex trade-off. Manufacturers commonly add high amounts of sodium, fats, and stabilizers to enhance texture and flavor, which can result in classification as ultra-processed (183). This is referred to as the health-sustainability-sensory trilemma: while aiming for sensory excellence, the nutritional profile that many consumers demand for sustainable alternatives could be upset. Moreover, the environmental impact from these proteins is much smaller than for conventional livestock products, but these benefits are only partly compensated for by consumer dissatisfaction with the texture, flavor, or pleasure of eating them. Food material innovation is therefore essential to match the sensory properties with their nutritional value, to provide a food system where sustainable protein is not only accepted by the global population but preferred, replete with the sensory attributes expected when consuming animal protein (184).

### 7.5 Labeling, transparency, and trust

Labeling, production disclosure, and consumer trust are critical factors that define the relationship between sustainable proteins and food systems on a global level. Nomenclature is a key psychological signal for consumers, especially when buying into new categories like cultured meat and insect food. Terms like lab-grown or synthetic often trigger associations with unnaturalness and technological interference, which can significantly diminish purchase intent (173). In contrast, descriptors like cultivated or cell-cultured are less emotive and more scientific, typically leading to increased acceptance as they support the idea that this is a controlled evolution of food production, instead of a radical break with nature (181). It is important to note that consumers often consider the naturalness and reduced processing of protein alternatives more important when assessing products, making this language relevant. Transparency about production methods serves as a prerequisite to institutional trust, particularly in markets where safety and ethical issues remain prominent. In the case of cellular proteins, radical transparency that integrates open communication about culture media, the elimination of animal slaughter, and rigorous safety measures is vital to counteract skepticism common in emerging markets (182). However, transparency is also crucial in the plant-based market, as consumers are becoming more aware and cautious about additives and the use of highly processed plant matter. Educating consumers about ingredients and their benefits has been demonstrated to positively impact purchase intent and brand retention over time (185). Moreover, institutional trust is paramount; consumers are more willing to accept novel proteins if they are confident in the regulatory authorities that ensure food safety and the companies that innovate new proteins.

Labeling is a vital part of bridging the gap between complicated production and well-informed buying decisions, but it faces challenges with regional regulations. Strategic labeling should aim to provide accurate information without causing uncomfortable sensory or psychological feelings. In Western nations, subtle front-of-package labeling has been used to remind consumers of the product format (e.g., protein-enriched pasta) instead of the biological source, to lower the disgust factor and foster familiarity over time (185). Eco-labels and carbon footprint indicators provide valuable environmental information but are usually secondary in the hierarchy of consumer decisions. Currently, consumers face difficulty with labeling due to the lack of standardized labeling across different jurisdictions, which can lead to confusion and a loss of trust in the product depending on the claims made. Finally, a holistic approach to communication will be needed to bridge the gap between the technology of sustainable proteins and the values of the contemporary consumer, including standardized terminology, educational campaigns, and clear labeling (173).

### 7.6 Cultural and regional perspectives

The psychological barrier of food neophobia, or the aversion to eating new foods, is also highly variable depending on the local food patrimony, adding further regional differences (186). Although the world strives for sustainability, research in Saudi Arabia shows that many older people and women have high neophobia and view some novel proteins as unclean, hindering sustainability efforts (187). In contrast, the Chinese are very sensitive to the linguistic framing of products: those with natural or traditional culinary cues are clearly preferred over those with technical or artificial terms. Furthermore, in multiethnic Asian populations, the acceptance of cultured meat and plant-based alternatives is often mediated by a complex interplay of ethnic identity and exposure to global food trends. Religious beliefs represent one of the most important regional barriers to the acceptance of biotechnology proteins. Cultured meat will require Halal and Kosher certification in markets with significant Muslim or Jewish consumer bases (188). Muslim consumers in the UAE have been seen as more hesitant to eat cultured meat, with some stating that the production processes need to comply with Islamic dietary requirements and be considered a solution to the country's food security. This shows the need for a successful protein transition to be localized to accommodate the religious and ethical frameworks already in place worldwide (189). Overall, understanding these regional nuances is key to ensuring that innovative protein technologies are not just a mass market phenomenon, but are also adapted to fit local tastes, religious beliefs, and language requirements, to name a few factors, for use in product development and marketing strategies.

Social norms, encompassing implicit norms (unstated cultural expectations), explicit norms (openly communicated social approval or disapproval), and dynamic norms (information that a behavior is increasingly being adopted by others), represent an underexplored but influential mechanism shaping protein-related food choices. Previous studies showed that communicating a dynamic norm (e.g., that meat reduction is becoming more common) can increase interest in and uptake of plant-based options even where the behavior remains a minority practice, by signaling an emerging social trend rather than simply describing current prevalence. Applying implicit, explicit, and dynamic norm framing across the cultural contexts discussed above represents a promising, currently underused strategy for accelerating consumer acceptance of sustainable proteins (190).

### 7.7 Willingness to pay and market segmentation

The commercial viability of sustainable proteins is inextricably linked to consumer willingness to pay (WTP) and the strategic identification of viable market segments. The environmental advantages of alternative proteins are often touted, yet recent empirical data indicates that the concept of WTP is multidimensional, and that a combination of sensory expectations, price thresholds, and institutional trust plays a part in this. For example, studies on cultured beef suggest that sustainability information can raise the mean WTP slightly, but sensory barriers, specifically issues of taste and naturalness, still pose a major hurdle. However, price is a clear barrier in specific markets such as Türkiye, where a clear price threshold could be identified that the majority of the population is unwilling to cross, even though they understand the sustainability of the product (191). Consumers are willing to pay a higher price for other novel foods than those derived from insects, depending not only on ecological or nutritional properties, but also on the recognizability of and trust in food safety standards associated with those products. This suggests that a more generic sustainability-based approach might not be enough to overcome the intention-behavior gap commonly encountered in the alternative protein space (192). Understanding the heterogeneity of consumers is important for market adoption, because a “one size fits all” strategy will not reflect the different motives behind dietary change.

Market segmentation research has revealed a number of consumer types in different contexts. In Spain, for instance, the market is divided into two categories: price-sensitive millennials and environmentally conscious consumers, as well as an indifferent group not particularly affected by the ethical marketing approach (193). Likewise, there is considerable variation in the WTP for plant-based and cell-based alternatives among segments in emerging markets such as India, which are often segmented by urban and income status (194). Demographic variables like gender and age further narrow down these segments, with middle-aged and middle-class people likely to be the main group to embrace cultured meat once prices are on par with conventional meat. Psychographic profiles (PSS), especially food neophobia, are also important predictors of acceptance, differentiating early adopters from late majority segments (191). Stakeholders need to match product features (e.g., sensory properties and competitive price) with the needs of the identified segments to achieve faster market adoption. Strategies that focus on sensory engagement through tasting activities and subtle labeling seem to be more successful than any overt technological framing. Finally, consumers' behavior change stage can be used to profile them and develop targeted interventions that can help make sustainable proteins a more common part of their diet (195).

## 8 Economic viability and regulatory considerations

### 8.1 Production costs and economic feasibility

Many fundamental constraints have hindered the transition to sustainable protein systems to date, the most prominent of which is the economic viability of alternative proteins compared to conventional animal proteins. The economic maturity and cost parity paths are very unevenly distributed across different protein categories. Microbial proteins, especially SCP from gas fermentation or agro-industrial waste, have great potential for the near future. For example, methanotrophic bacteria, which use landfill or wastewater treatment gas, can achieve production costs in the range of US$1,600 per metric ton, which is competitive with fishmeal. These processes are very sensitive to electricity pricing and demand, as energy-efficient bioreactor cooling and biomass drying are significant operational costs (196). The integrated circular economy model, which involves using cheese whey for fermentation, can lower this cost even further to €5 per kg, offering better protein productivity than soybean meal (197). More established alternatives such as mycoprotein, which is made by the fermentation of fungi such as *Fusarium venenatum*, already have the potential to become price competitive with beef on a per-protein basis. Mycoprotein is competitive in price with high-value red meat but is not as affordable as commodity chicken or ingredients for pet food from cheap animal by-products (198). By contrast, cultured meat or animal cell-based meat (ACBM) is the most economically challenging. Recent techno-economic analysis shows that ACBM is still very costly for mass production because of the high initial investment and specific technical requirements. The price of growing media such as pure amino acids and growth factors continues to be a major cost, but plant-based hydrolysates are being tested as a more economical alternative (199). Furthermore, metabolic limitations, such as low cellular growth rates and shear-induced damage, restrict the attainable cell density in large-scale bioreactors, preventing the industry from reaching the theoretical performance limits needed to displace conventional meat commodities (200). Scaling up innovations and optimizing input costs are essential to the financial viability of sustainable proteins. Though microbial and fungal proteins are catching up with animal-based benchmarks, cultured meat is restricted to niche high-value markets until there are significant advances in bioprocess engineering and media formulation. Additional optimization of fermentation pathways and identification of low-cost, sustainable substrates will be needed for long-term adoption, as they will guarantee a sustainable and economically competitive future food system (201).

### 8.2 Scaling challenges

While sustainable proteins are transitioning from small-scale research to a global market, substantial technical and economic challenges remain. The most current challenge is high production costs, with many alternative protein sources, especially cultured meat and insect-based proteins, not yet achieving the manufacturing efficiency needed for cost-competitiveness with regular animal products (160). Although the plant-based sector is more developed in its industrial processes, there is a significant “valley of death” in the field between laboratory innovation and large-scale industrialization (162). Reaching economies of scale requires huge investment in specialized infrastructure, including high-capacity bioreactors in cellular agriculture and automated rearing facilities for entomophagy (202). Furthermore, current manufacturing practices suffer from high energy consumption and low yields, necessitating breakthroughs in synthetic biology and metabolic modeling to improve protein expression and resource conversion rates. In addition to economic considerations, several technical challenges impede market expansion, including the functional and sensory characteristics of these proteins. Alternative proteins should not only have a sustainable production method, but also possess a texture, flavor, and nutritional value comparable to that of meat to achieve long-term consumer acceptance (203). Large-scale production validation for continuous processes has proved challenging at the formulation level due to the biochemical activity of new proteins being affected by scale-up. Such technical variability can lead to inconsistencies in product quality, potentially harming consumer trust and brand loyalty. Moreover, the implementation of such novel ingredients into current global food systems must involve the creation of novel supply chain solutions that will guarantee traceability and safety-by-design (204).

The regulatory environment adds complexity to scaling up as it is inconsistent across countries, leaving investors and producers unclear about the regulatory landscape. In many countries, novel foods are not legally defined, and safety assessment procedures are still being developed. The regulatory process for cultivated meat involves complex assessment of the stability of cell lines and media ingredients, with significant differences between regulatory agencies (202). This uncoordinated regulatory framework impedes cross-border trade and delays the deployment of global production networks. To address these challenges, greater industry-academy-governmental cooperation is needed to create comprehensive safety regulations and standardized guidelines. Lastly, the socio-economic consequences of scaling sustainable proteins are not insignificant: a swift transition to alternative proteins could have a disruptive effect on traditional farming livelihoods and land-use practices that need to be addressed with proactive policies to ensure a just transition for traditional farmers (205).

### 8.3 Food safety and risk assessment

Food safety and risk assessment are the pillars of integrating sustainable proteins into the food system without unintended consequences for public health. With the global shift toward alternative protein sources (plant-based, insect-based, cultured meat, and microbial-derived), stringent hazard identification and mitigation measures are necessary (206). Microbiological safety remains a major consideration across all these different fields. Insect foods are typically free of zoonotic diseases, but there are potential risks such as food-poisoning pathogens, parasites, and viruses, which are strongly correlated with rearing substrates and processing conditions (207). The application of strict hygienic preventive measures and specific decontamination measures, including targeted heat treatment, is key to reducing these risks and providing product stability. Likewise, plant-based proteins are exposed to microbiological risks during harvesting and processing, and also potentially to contamination by chemicals, such as heavy metals or pesticide residues, which can bioaccumulate in the plant based on soil quality (208). For technologically advanced industries such as cultured meat and microbial proteins, chemical safety assessment is especially complex. In cultured meat production, the growth media consists of a combination of factors and hormones, and residues in the final biomass should be carefully and systematically monitored to avoid exceeding safe limits. High-sensitivity analytical methods, such as liquid chromatography-tandem mass spectrometry (LC-MS/MS), have recently been used to quantify low levels of risk substances such as forskolin and thymidine in cell biomass, showing that successive washing steps can decrease these substances to below detection limits (209). The potential for microbial production of mycotoxins and the safety of the microbial production strains must be confirmed by extensive toxicological testing prior to commercial use of microbial proteins, especially those from specialized fungi or yeast strains. Another important risk factor is allergenicity, particularly if the novel proteins are cross-reactive with other allergenic proteins. Crustacean and house dust mite proteins are often also found in insect proteins, requiring specific warning statements to be mandatory on the product label for those who are sensitive (210). Moreover, many of these microbial and cell-based proteins are novel and need rigorous allergenicity testing and post-marketing surveillance to identify new adverse reactions. Regulatory frameworks like Novel Food Regulation (EU) 2015/2283 and the FDA consultation process ensure oversight by demanding comprehensive dossiers covering production methods and toxicological data. Responsible innovation frameworks are, however, needed in the industry, which incorporate multi-criteria decision analysis and give attention to balancing nutritional benefits with hazards (211). In conclusion, the need for standardized analytical methods and reporting is crucial for consumer trust and the viability of these systems.

### 8.4 Regulatory frameworks across regions

The regulatory landscape for sustainable proteins is characterized by significant regional divergence, reflecting varying institutional priorities, legal traditions, and economic strategies. In the United States, the FDA and the U.S. Department of Agriculture (USDA) share specific regulatory responsibilities concerning novel protein sources such as cultivated meat. This dual-agency approach puts the FDA in charge of the cell banking, cell collection, and cultivation stages of the production cycle, while the USDA takes over when the cells are harvested, processed, and labeled (212). The U.S. does not have a specific novel food classification for these products, but rather relies on existing food safety frameworks applied to alternative food production processes, such as using the “Generally Recognized as Safe” (GRAS) notification system for plant-based ingredients. This enabled the pioneering commercial approvals of cultivated chicken in late 2022 and early 2023 (213). The European Union has a very centralized and precautionary strategy, managed by the Novel Food Regulation (EU) 2015/2283. In this framework, all food products not substantially used in the Union prior to May 1997 are required to undergo a thorough pre-market safety evaluation by EFSA. While the process for cultivated meat is more complex than for certain insect species successfully authorized under this framework, it offers a strong scientific pathway toward sustainable proteins. The EU system requires applicants to prove that the product is safe, not nutritionally disadvantageous, and labeled accurately to avoid misleading consumers. The authorization procedure is reportedly lengthy and politically influenced by member states, leading to the absence of any cultivated meat products on the EU market yet (214). The Asia Pacific region is characterized by a dynamic and increasingly proactive regulatory environment, with Singapore being a global pioneer. To enable the first commercial approval of cultivated meat, the Singapore Food Agency (SFA) developed a novel food framework focused on safety-by-design principles and Good Cell Culture Practices (GCP) in 2020. Extensive interactions with international scientific committees and emphasis on Hazard Analysis Critical Control Points (HACCP) in bioreactor scale production support Singapore's regulatory agility (215). Food Standards Australia New Zealand (FSANZ) is using established food standards to assess alternative proteins, but manufacturers may face uncertainty around newer technologies such as microbial biomass. In parallel, synthetic meat and alternative proteins have been officially included in China's 14th Five-Year Agricultural Development Plan, a clear strategy to step up its regulatory support and domestic scaling efforts in the near future (203). Overall, the regional variation contributes to a complicated global market in which biotechnological innovations are frequently outpacing the creation of globally accepted safety and labeling standards.

### 8.5 Investment trends and industry development

Transition to sustainable protein systems is in a state of profound structural transformation of investment paradigms and industrialization. Traditionally, the alternative protein industry (plant-based, precision fermentation, and cultivated meat) has been supported by VCs, especially during the initial stages of product development. As these technologies become more viable for commercialization, however, the investment environment is becoming more stable and long-term, with money now coming from sovereign wealth funds, institutional investors, and traditional food industry participants. This turn is crucial to overcome the often-cited valley of death for capital-intensive biological start-ups that involves high capital costs for industrial production (216). One particularly difficult aspect of scaling precision fermentation for dairy proteins is that a significant capital investment is required to expand production capacity, but this investment is often deferred until larger-scale feasibility is demonstrated. In response to this, government intervention has come to be a staple of industry development. Market barriers like high production costs and regulatory uncertainty are overcome through the use of public policy and strategic research funding. Other regions, such as Singapore, have created strong institutional structures specifically dedicated to developing alternative protein farming through coordinated research programs and favorable policies (217). Likewise, countries such as China are encouraging domestic plant-based meat manufacturing, offering policy guidance that reduces the price premium for plant-based proteins compared to conventional ones. This institutionalization of the sector signals a transition from speculative innovation to a permanent fixture of global food security strategies. The industry is also experiencing major consolidation as it transitions from experimentation in the pilot stage to production at a much larger scale. Although plant-based meat is the most developed category, the cultivated meat and precision fermentation categories are both engaged in efforts to make their products cost-competitive with animal-based meat, requiring not only technological optimization but also the establishment of specialized supply chains for raw materials and culture media (218). The level of market maturity is also reflected in the growing number of meat processors entering the market by either buying start-ups or establishing their own alternative protein businesses to diversify and reduce environmental risks. It also poses challenges around power dynamics and the possibility of traditional actors “hijacking” the sustainability narrative (219). Additionally, with the upsurge in production, the industry is improving its production focus on nutritional fortifications and sensory improvement for wider consumer acceptance. Additionally, the economic potential of sustainable proteins is contingent on a balance between private innovations, public investment in infrastructure, and the establishment of a common regulatory framework for global markets (204). Regulatory status and commercialization readiness of emerging protein technologies are summarized in Table 3.

**Table 3:** Regulatory status and commercialization readiness of emerging protein technologies.

Protein technology	Regulatory status	Commercialization readiness	References
Plant-based analogs	Generally recognized as safe (GRAS); established regulatory frameworks	High; widely available in global retail and food service	(5)
Mycoproteins	Approved in Europe and several global regions; established safety track record	Moderate to high; production is scaling with global distribution	(134)
Insect protein	Variable by region; requires Novel Food authorization in the EU	Emerging; limited by cultural acceptance and current production scale	(247)
Cultured meat	Under development; requires rigorous policy frameworks and safety approvals	Low; dependent on technological breakthroughs for cost-effective scaling	(5)
Microbial proteins	Pending comprehensive regulatory assessment for wider food applications	Early stage; primarily in research and pilot-scale development	(25)
Microalgal protein	Generally Recognized as Safe for specific strains; ongoing safety analysis	Emerging; increasing inclusion in specialized health and food products	(248)

## 9 Technological innovations driving sustainable protein development

### 9.1 Artificial intelligence and machine learning

Artificial intelligence (AI) and machine learning (ML) have become key enablers for sustainable protein systems in the digital age, providing unparalleled rapidity and accuracy in product development. The technologies tackle the fundamental barriers to alternative proteins: nutritional value, sensory similarity to animal protein, and bioprocess scalability. The realm of discovery has recently seen the application of transformer-based protein language models (pLM) to predict the true ileal digestibility coefficient of novel plant proteins with up to 90% accuracy, effectively cutting out the need for extensive *in-vivo* animal testing. These predictive systems can help researchers quickly scan large botanical libraries for proteins that meet certain nutritional needs, such as proteins specifically designed for pediatric nutrition, where digestibility is a concern and natural bitterness needs to be minimized (220). Besides discovery, ML methods are also important in improving the sensory profiles used to assess consumer acceptance. Advanced deep learning models have the ability to intelligently decompose complex flavor and texture characteristics to produce alternative protein products that closely match traditional meat (221). Bayesian Optimization (BO) has been more successful than Response Surface Methodology (RSM) in optimizing plant-based protein extrusion. This method uses probabilistic surrogate models to converge with fewer experimental trials and maintains a small prediction error on mechanical properties (such as tensile strength) of meat analogs, while preserving structural integrity (222).

Texture, a key consumer challenge, is now being examined with the aid of AI-generated mechanical profile analysis, allowing for continuous enhancement of alternative protein burgers. AI systems are particularly vital in cultivated meat biomanufacturing, notably in quality control and scalability. In large-scale cultured meat production, a large number of cells are utilized to monitor their status and the quality of cell passages, including Hanwoo cattle satellite cells. These digital interventions control complex bioprocesses that are more likely to be cost-effective for industrial use (223). Specifically, artificial intelligence has helped to create microbial optimization, making precision fermentation much more efficient. When this reinforcement learning/genetic engineering approach is combined with CRISPR technology, it yields up to 300% more alternative proteins and reduces bioreactor failure by 60%. The use of advanced calculation techniques such as artificial neural networks (ANN) in conjunction with genetic algorithms (GA) provides higher statistical reliability for predicting optimal media concentrations than conventional statistical methods (36). Lastly, the use of multi-objective reinforcement learning for mycoprotein fermentation has led to an additional gain of 10%−20% protein yield and significantly lowered downstream processing costs (220). These AI and ML innovations, when considered together, offer the backbone of a more resilient and efficient food system of the future.

### 9.2 Omics technologies

The advent of omics technologies (genomics, proteomics, metabolomics) has proven essential in pushing sustainable protein development forward and becoming an indispensable tool in bridging the gap between biological potential and consumer expectations. At the genetic level, genomics lays the groundwork for optimizing protein sources, which can be used to develop improved crops and engineered microbial strains (40). Within plant-based proteins, genomic selection and molecular breeding are applied to identify germplasm characteristics and to map fundamental desired traits, including protein content and anti-nutritional compounds, enabling the development of better varieties for future food systems. PF and cell-based meat can rely on high-yield microbial strains and robust cell lines developed by whole-genome sequencing and CRISPR editing to ensure scalability and effectiveness for biomanufacturing (224). In addition to this genetic information, proteomics provides detailed insights into the composition and behavior of proteins, essential for replicating the texture and mouthfeel of animal-based products. Advanced mass spectrometry techniques can be used to trace changes in structure and interactions that influence key characteristics such as solubility, gelling, and emulsification. The proteome of plant-based ingredients can be profiled to detect specific peptides that may affect digestibility, while others may contribute to off-flavors, and targeted processing interventions can be undertaken to enhance the sensory profile (225). Moreover, metabolomics is essential to understanding the chemical complexity of sustainable proteins, especially concerning their flavor and nutrient completeness. Non-targeted metabolomic profiling can be used to map amino acids and organic acids involved in umami intensity and the stability of the overall taste in fermented and cultivated foods (226). This technology is also crucial for bioprocess monitoring, as it provides immediate information on metabolic pathways during fermentation, guaranteeing product quality and ensuring safety (227). By combining these multi-omics technologies, a comprehensive picture of alternative proteins can be gained, overcoming major challenges including allergenicity, nutrient bioavailability, and environmental trade-offs. Through optimizing the structural and chemical characteristics of these proteins, omics technologies, when directly applied to consumer acceptance, boost palatability and nutritional parity with traditional meat. Ultimately, the organized use of omics data, often complemented by artificial intelligence, contributes to the development of culturally appropriate, sustainable, and high-quality food solutions for global populations, tailored to each person's specific needs (40).

### 9.3 Synthetic biology

Synthetic biology has become a transformative technology for the pursuit of sustainable protein sources, providing a programmable method of food production beyond the boundaries of conventional agriculture. It is essentially a metabolic engineering and rational design approach that repurposes microbes such as yeast, fungi, and bacteria as efficient biological factories for the production of complex proteins. One of the major applications is PF, which enables the production of animal-identical proteins such as collagen, gelatin, whey, and casein without the use of animals (228). These bio-identical ingredients are crucial for replicating the sensory and functional characteristics of conventional animal products, such as thermal reversibility and specific mouthfeel, which are often difficult to achieve with plant-based alternatives alone. Microbial hosts can be engineered to synthesize proteins with 95%−99% sequence similarity to the protein's native animal species, allowing for both nutritional equivalence and consumer recognition (229). These innovations are further driven by the incorporation of artificial intelligence and bioinformatics, allowing for the design of biosynthetic pathways using a model-based approach. The design-build-test-learn process enables customization, the creation of nutrient-rich proteins with specific amino acid compositions, and the programming of sensory properties for specific applications to meet the dietary needs of different human populations (230). In addition to improving food quality, synthetic biology is also a powerful tool for environmental mitigation. PF technologies are expected to cut dairy and meat alternative GHG emissions by as much as 97% and land use by 90% compared to conventional meat production. Furthermore, synthetic biology empowers the development of carbon recycling as a protein production route by engineering microbes to use C1 compounds and waste streams as feedstocks, thus enabling a shift toward a circular bioeconomy (231).

However, the path toward mainstream adoption is not without hurdles. Process intensification and scalability are still required to decrease the production cost of recombinant proteins compared to traditional dairy and meat products. Strategic areas of focus include applying AI metabolic modeling to optimize bioprocessing and reduce expenses, and navigating intricate regulatory frameworks around the world. Lastly, consumer acceptance of synthetic and fermentation proteins is key: they must be safe, natural, and sustainable for consumer acceptance in future food systems (232). The future evolution of resilient and sustainable global protein sources will remain on track with the continued advancement of synthetic biology, machine learning, and omics (228).

### 9.4 Precision fermentation advances

Precision fermentation (PF) is a revolutionary technology that involves the use of engineered microorganisms as cell factories to produce specific functional ingredients. Recent advances focus on metabolically engineering host strains like Saccharomyces cerevisiae, Pichia pastoris, and Trichoderma reesei using CRISPR-Cas9 and AI-driven bioprocessing to increase protein titers and yield efficiency (233). These advancements have led to the creation of animal-like proteins such as the milk proteins whey and casein, which share over 95% sequence identity with the proteins present in milk, and egg proteins such as ovalbumin, which shares a sequence identity of between 95% and 99% with its natural counterpart (229). These recombinant proteins can be used to impart complex functional properties to plant-based ingredients, such as gelling, foaming, and emulsification. This enables their perfect integration into existing food matrices and offers the same essential amino acid profile and nutritional quality as their animal-derived counterparts (234). From an environmental perspective, precision fermentation offers a significant reduction in resource intensity compared to conventional livestock agriculture. According to life cycle assessments, PF can lower greenhouse gas emissions by as much as 97%, land use by about 99%, and water use by 99.7%. The net sustainability of such systems, however, strongly depends on the energy supply mix in the production region and the sources of carbon feedstocks, and requires transition toward renewable energy and circular bioeconomy feedstocks to maximize ecological benefit (235). However, there are significant economic and social challenges to adapting to a protein-rich environment. Recombinant protein production costs are still much higher than for conventional bovine proteins, at $210 to $310/kg, vs. about $15 to $25/kg for conventional bovine proteins. To achieve price parity, the scale of fermentation will need to increase substantially, and industry goals call for titers of more than 50 g/L in bioreactors of over 100,000 liters. Moreover, regulatory uncertainties surrounding novel foods and consumers' doubts about GM products are still important for broader market entry (236). PF holds promise as a key component of the future resilient food system, which can continue to be developed by technological advancements that will further reduce costs and enhance scalability, avoiding the ethical and environmental burden of industrial animal farming (229).

### 9.5 Smart manufacturing and industry 4.0

The Fourth Industrial Revolution, which is bringing together physical, digital, and biological systems, is changing the face of alternative protein production. With unprecedented pressures on the global food system to provide sustainable and quality nutrition, smart manufacturing and Industry 4.0 have become key enablers in the development of plant-based analogs, cultured meat, and microbial proteins (9). These technologies allow for the transition from batch production to smart, self-optimizing systems that are more resource-efficient and product-consistent. These changes involve the adoption of artificial intelligence (AI) and machine learning, now widely used to simulate complex biochemical reactions and adapt production conditions on the fly (237). For the plant-based category, computational modeling using AI can help determine new ingredient combinations, identify structural changes occurring during processing, reduce the environmental footprint of product development, and enhance organoleptic properties (226). In addition to these digital analytical tools, the Internet of Things (IoT) provides an integrated solution to track all elements of the production process (238). Smart sensors are deployed throughout the manufacturing floor to track energy consumption, water, temperature, etc., to ensure that cultivation and fermentation processes remain within a safe and quality range. In the case of cultured meat, Industry 4.0 technologies play a key role in resolving the biological and technological challenges of scaling up production in large-scale bioreactors (239). Digital twins can be used to generate virtual copies of physical bioreactors, which can simulate various situations and help operators adopt predictive maintenance practices to avoid failures in their batches. Moreover, the precise manipulation of microstructures in alternative proteins, made possible by additive manufacturing, particularly 3D printing, has revolutionized texturization, enabling the mimicry of the fibrous texture of meat and seafood (249). The implementation of these smart manufacturing systems is also crucial in promoting consumer acceptance by providing transparency and traceability in the supply chain. Through data-driven insights, manufacturers can offer substantiation on the sustainability and nutritional attributes of their products and respond to consumer concerns about novel food technologies (250). The potential cost savings afforded by automation, advanced analytics, and biotechnology are likely to be substantial as biomanufacturers continue to embrace these Industry 4.0 principles, ultimately allowing sustainable proteins to achieve parity with traditional animal proteins. In conclusion, the integration of smart manufacturing into the alternative proteins industry holds significant potential for contributing to a more resilient and circular global food system that can support the nutritional needs of a growing population while preserving the health of the planet (237).

### 9.6 Digital traceability and sustainability monitoring

The use of Industry 4.0 technologies is changing how the alternative protein supply chain is verified and managed, enabling the traceability and management of sustainable claims with immutable data. Digital traceability, particularly through blockchain technology, has become a foundation for fostering consumer trust in emerging protein sources like plant-based, insect-based, and cultured proteins (9). Blockchain ensures that every stakeholder, from raw materials producers to the end consumer, has a verifiable and transparent history of a product's journey (251). This is especially important when making “green” claims for alternative proteins and confirming that there are no conventional animal-derived contaminants. The combination of blockchain and the Internet of Things (IoT) takes sustainability monitoring beyond auditing, with real-time data tracking capabilities. Energy consumption, water consumption, and carbon emissions can be continuously recorded with IoT sensors deployed in production facilities or during transportation (252). In precision aquaculture, IoT-powered growth trackers and cryptographic algorithms enable the remote monitoring of protein sources such as mussels to ensure data integrity and maximize resource efficiency. These automated solutions minimize the risk of human error and data manipulation, deliver a high-fidelity LCA, and allow consumers to interact directly with the system by using smart labeling or QR codes (253).

Furthermore, AI and knowledge graphs can help to intelligently analyze the huge volume of data generated by these digital tools. AI can identify inefficiencies in the supply chain, suggest improvements for reducing the environmental footprint of protein alternatives, and even predict potential safety problems before they reach consumers (9). Such technological platforms also play an important role in preventing food fraud as they provide a robust framework for authentication verification. Digital traceability will enable detailed insight into the production process, from the fermentation parameters for mycelium-based proteins to the nutrient composition of insect flours, making it not just a possibility or theory, but a definite reality (254). These digital infrastructures will be vital for long-term transparency to drive the transition of the global food system toward more sustainable protein alternatives (251).

## 10 Future perspectives and research priorities

The transition to sustainable protein systems needs to move beyond safety assessments for individual products to integrated, whole-of-chain assessments that reflect the complexity of global food systems.

One of the main research areas is the harmonization of sustainability measures, including the standardization of LCA approaches. The environmental impact of novel sources, such as cellular agriculture or microbial proteins, relative to traditional agriculture is currently difficult to measure because of the lack of integrated data and boundaries. A common reporting framework will be essential to ensure meaningful, measurable, and global access to reporting information that can guide industry actions and consumer choices (255). Beyond environmental footprints, nutritional standardization is a critical frontier. The industry should implement universal indicators of protein quality such as the PDCAAS or DIAAS to address the increasing dietary protein requirement of a growing and aging population and guarantee food security in this context (2). Future research should focus on the digestibility of the protein in alternative protein sources, as they may be less digestible than animal protein. The complex nutritional requirements of different populations demand innovative processing and production techniques like precision fermentation and ingredient blends to maximize the nutritional and functional attributes of alternative proteins (256). Long-term health effects of using meat alternatives are not well studied, and future research should further examine their use beyond safety. These studies should examine the human microbiome and chronic disease indicators over the course of decades with sustained consumption of highly processed plant-based or lab-grown proteins. At the same time, consumer behavior science needs to focus on the psychological and cultural resistance that stands in the way of the mass adoption of new proteins (257). In practical terms, this means behavioral and consumer scientists, in collaboration with food technologists and public health nutritionists, should prioritize intervention research (e.g., framing, labeling, and repeated exposure strategies) and cross-cultural comparative studies that move beyond documenting barriers to testing scalable strategies for shifting the purity-related and naturalness-based objections identified in the consumer acceptance literature (258), with findings intended to directly inform product developers, regulators, and public health communicators on which specific interventions to prioritize and for which populations.

It is vital to establish trust by communicating openly about the health benefits and technological safety of the technology. To transform alternative proteins from niche to mainstream use, it will be essential to gently persuade consumers to shift toward alternative protein sources while maintaining respect and appreciation for their culture. Circular economy approaches can be a viable pathway for resource efficiency in the protein supply chain. A systematic upcycling of agricultural side-streams and food wastes into high-value protein ingredients, including the use of insect meal or single-cell proteins, should be given high research priority (259). This approach optimizes land and water usage and enables waste to become part of a resilient food system. However, these technologies require addressing for scaling with strong policy support. Governments must coordinate or harmonize subsidies, labeling requirements, and safety-certification procedures to create a level playing field for sustainable alternatives. Flexible regulatory systems are needed to accommodate rapid technological change without compromising costs and access, particularly in low- and middle-income areas (260). The future of global food systems should be resilient to feed the world's population of 9 billion by 2050 in the context of increasing pressures from climate change. This change needs to be systemic, with models of agroecology that integrate plant and animal proteins and a One Health approach (261). Sustainability, profitability, and nutrition should be considered as three connected pillars in future protein systems. To develop a sustainable and nutritious protein landscape, it is necessary to act transsectorally and with a long-term research approach (255).

## 11 Conclusion

The transition toward sustainable proteins represents a critical cornerstone in the global strategy to address the intersecting challenges of food security and environmental degradation. As this review underscores, the potential for alternative protein sources, encompassing plant-based, insect-derived, microbial, and cultured varieties, to mitigate the ecological pressures of traditional livestock is immense. However, their successful adoption is not without significant complexities. Nutritional quality varies substantially across these novel sources, necessitating a rigorous and standardized evaluation of amino acid profiles and bioavailability to prevent unintended public health outcomes. Furthermore, while the environmental advantages are significant, they must be meticulously weighed against the energy-intensive processing trade-offs inherent in many technological innovations. Beyond technical feasibility, consumer acceptance remains the ultimate determinant of market viability, requiring targeted strategies to overcome psychological and cultural barriers. To effectively scale these solutions by 2050, the integration of circular economy principles, sustained technological advancement, and supportive multi-sectoral policies will be indispensable. Moving forward, the global protein landscape must be evaluated through holistic and integrated frameworks that synthesize nutritional, environmental, economic, and social sustainability metrics. Only through such a comprehensive approach can we build a resilient and equitable food system capable of nourishing a growing global population within planetary boundaries.
